# Conditional mouse models support the role of SLC39A14 (ZIP14) in Hyperostosis Cranialis Interna and in bone homeostasis

**DOI:** 10.1371/journal.pgen.1007321

**Published:** 2018-04-05

**Authors:** Gretl Hendrickx, Vere M. Borra, Ellen Steenackers, Timur A. Yorgan, Christophe Hermans, Eveline Boudin, Jérôme J. Waterval, Ineke D. C. Jansen, Tolunay Beker Aydemir, Niels Kamerling, Geert J. Behets, Christine Plumeyer, Patrick C. D’Haese, Björn Busse, Vincent Everts, Martin Lammens, Geert Mortier, Robert J. Cousins, Thorsten Schinke, Robert J. Stokroos, Johannes J. Manni, Wim Van Hul

**Affiliations:** 1 Center of Medical Genetics, University and University Hospital of Antwerp, Antwerp, Belgium; 2 Department of Osteology and Biomechanics (IOBM), University Medical Center Hamburg-Eppendorf, Hamburg, Germany; 3 Center for Oncological Research Antwerp (CORE), University of Antwerp, Antwerp, Belgium; 4 Department of Otorhinolaryngology, Radboud University Medical Center, Nijmegen, The Netherlands; 5 Department of Periodontology and Oral Cell Biology, Academic Center of Dentistry Amsterdam and VU University Amsterdam, Amsterdam, The Netherlands; 6 Food Science and Human Nutrition Department and Center for Nutritional Sciences, College of Agricultural and Life Sciences, University of Florida, Gainesville, FL, United States of America; 7 Department of Neurosurgery, University Hospital Antwerp, Antwerp, Belgium; 8 Department of Pathophysiology, University of Antwerp, Antwerp, Belgium; 9 Department of Pathological Anatomy, University Hospital Antwerp, Antwerp, Belgium; 10 Department of Otorhinolaryngology and Head & Neck Surgery, Maastricht University Medical Center, Maastricht, The Netherlands; Murdoch Childrens Research Institute, AUSTRALIA

## Abstract

Hyperostosis Cranialis Interna (HCI) is a rare bone disorder characterized by progressive intracranial bone overgrowth at the skull. Here we identified by whole-exome sequencing a dominant mutation (L441R) in *SLC39A14* (*ZIP14*). We show that L441R ZIP14 is no longer trafficked towards the plasma membrane and excessively accumulates intracellular zinc, resulting in hyper-activation of cAMP-CREB and NFAT signaling. Conditional knock-in mice overexpressing L438R *Zip14* in osteoblasts have a severe skeletal phenotype marked by a drastic increase in cortical thickness due to an enhanced endosteal bone formation, resembling the underlying pathology in HCI patients. Remarkably, L438R *Zip14* also generates an osteoporotic trabecular bone phenotype. The effects of osteoblastic overexpression of L438R *Zip14* therefore mimic the disparate actions of estrogen on cortical and trabecular bone through osteoblasts. Collectively, we reveal ZIP14 as a novel regulator of bone homeostasis, and that manipulating ZIP14 might be a therapeutic strategy for bone diseases.

## Author summary

Osteoporosis is a skeletal disorder affecting hundreds of millions of people, and is characterized by a low bone mineral density (BMD) and increased susceptibility to fracture. Genetic factors are the greatest determinants of BMD, but only a small fraction of these have been identified through genome-wide association studies. Studying rare, monogenic skeletal disorders is therefore an interesting strategy to identify genes with a putative large effect on BMD. Hyperostosis Cranialis Interna (HCI) is a rare monogenic disorder resulting in bone overgrowth exclusively at the skull, for which the underlying genetic cause was previously mapped to a region on chromosome 8. Our study demonstrates that HCI results from a mutation in *SLC39A14* (*ZIP14*), resulting in trafficking defects of ZIP14 and an aberrant cellular zinc homeostasis. Conditional mouse models demonstrate primary actions of Zip14 through osteoblasts, resulting in a HCI-like phenotype in the long bones and reveal estrogen-mimicking and PTH-opposing effects of Zip14 on bone homeostasis. This study designates ZIP14 as a novel regulator of BMD, and that manipulating ZIP14 might be a therapeutic strategy for complex bone diseases, like osteoporosis.

## Introduction

Hyperostosis Cranialis Interna (HCI, OMIM 144755) is a rare bone disorder characterized by endosteal hyperostosis and osteosclerosis of the calvaria and the skull base. This results in the entrapment and dysfunction of cranial nerves I, II, V, VII and VIII, causing disturbances in smell, vision, sensation in the face, facial expression, hearing and balance, respectively [1, 2]. In addition, increased ocular and intracranial pressure can occur, leading to frequent headaches. Remarkably, there is no indication that the remainder of the skeleton is affected in HCI patients. The first radiological abnormalities are often seen in the first decade, whereas the first symptoms occur late in the first decade or in adulthood and slow progression of the disease can be seen until the age of 40 [1, 2]. Untimely death may occur in severely affected cases, due to decreased intracranial volume [2, 3].

HCI was originally described by Manni *et al*. in three related families with common progenitors from the Netherlands with currently 13 affected family members over four generations [1]. This family is still the only family known with HCI. As a monogenic skeletal disorder, HCI has an autosomal dominant inheritance pattern. The genetic cause of HCI has been investigated previously by performing a whole-genome scan and linkage analysis in this family, where we assigned the locus for HCI to chromosome 8p21 [4].

The aim of this study was to further look for the disease-causing gene and get insights in the mechanism underlying HCI. Therefore, whole-exome sequencing was performed, which resulted in the identification of a missense mutation (p.L441R) in the *SLC39A14* (*ZIP14*) gene, encoding a zinc transporter. *In vitro* studies were performed to investigate the subcellular localization and p.L441R ZIP14. Furthermore, two conditional knock-in mouse models were generated, overexpressing p.L438R Zip14 in osteoblasts and osteoclasts. Thorough skeletal phenotyping of these mice was performed to unravel cell-specific effects of p.L438R Zip14 *in vivo*. Finally, to learn more about the pathogenesis of this disorder, histology of a HCI skull biopsy specimen was performed and luciferase reporter assays were done to look for aberrations in signaling pathways caused by p.L441R ZIP14.

## Results

### Identification of *SLC39A14* (*ZIP14*) as disease causing gene for HCI

**Fig 1:**
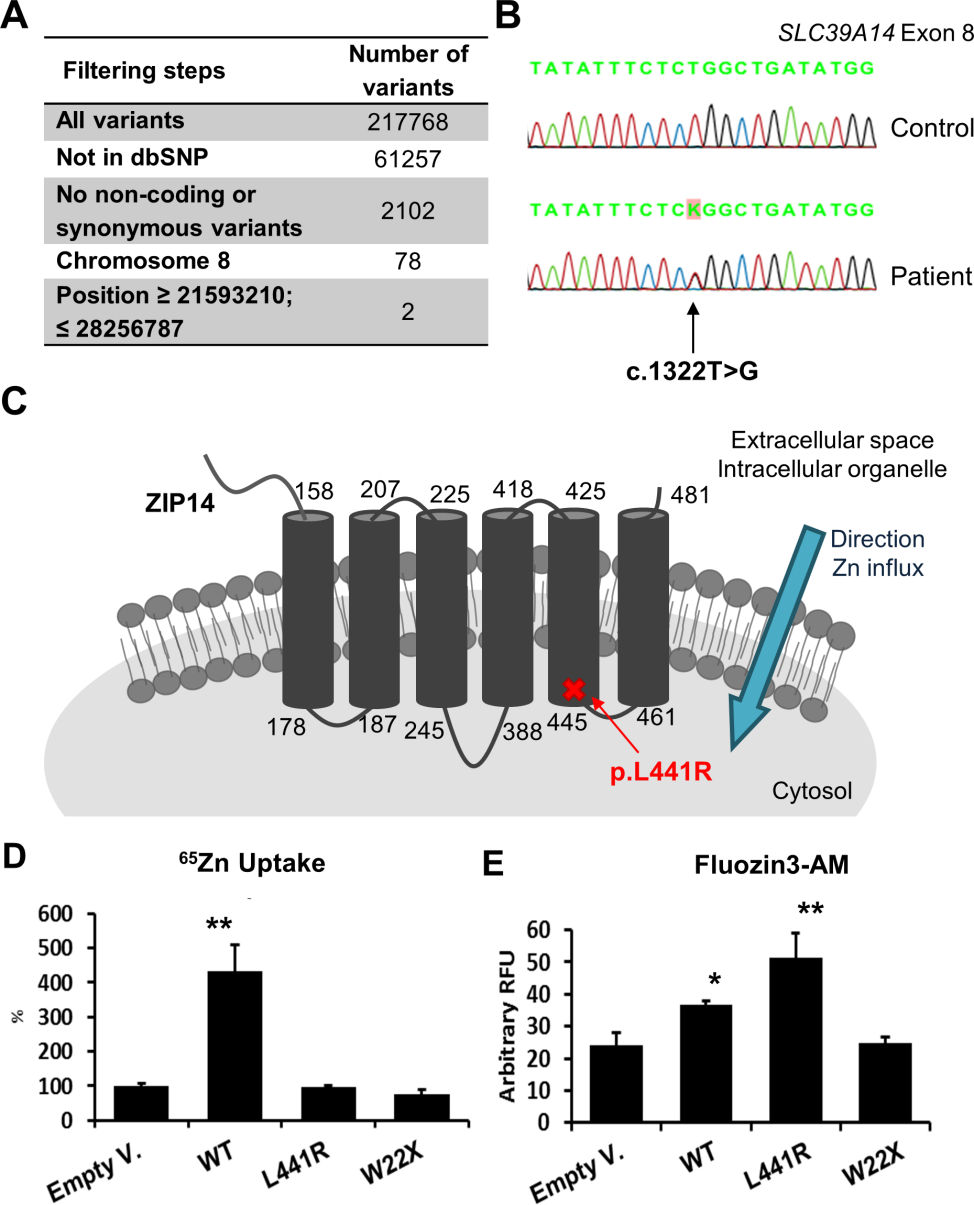
The c.1322T>G (p.L441R) mutation in *SLCA39A14* (*ZIP14*) was identified by WES and results in Zn uptake and accumulation defects. **(A)** Whole-exome sequencing (WES) was performed on one patient with Hyperostosis Cranialis Interna. Variants were filtered for their absence in dbSNP, by excluding non-coding and synonymous variants and its presence in the linkage region on chromosome 8 (chr8: 21,593,210–28,256,787) after which only two variants remained. **(B)** Identification of the c.1322T>G mutation in exon 8 of the *SLC39A14* (*ZIP14*) gene by Sanger sequencing. **(C)** Localization of the p.L441R mutation in the fifth transmembrane domain of ZIP14. **(D)** ^65^Zn uptake experiments demonstrate that WT ZIP14 significantly (*p*<0.001) increases ^65^Zn uptake when compared to cells transfected with empty vector (Empty V.). L441R and W22X ZIP14 show no sign of ^65^Zn uptake from the extracellular space into the cell. **(E)** FluoZin3-AM experiments demonstrate a significant (*p*<0.05) increase in Zn accumulation in cells overexpressing WT ZIP14. Overexpression of L441R ZIP14 results in a stronger (*p*<0.001) increase in intracellular Zn accumulation. *:*p*<0.05; **:*p*<0.001 by one-way ANOVA.

Whole-exome sequencing (WES) was performed on one affected individual from the family with HCI. The average coverage throughout the whole exome was 66x. After filtering variants for their absence in dbSNP and excluding non-coding and synonymous variants, we focused on the variants present in the linkage region on chromosome 8 (chr8: 21,593,210–28,256,787) after which only two variants remained (Fig 1A). Both variants, one in *SCARA3* with a 5x coverage and one in *SLC39A14* with a 66x coverage, were checked with Sanger sequencing. The variant in the *SCARA3* gene appeared to be a false positive, since we could not confirm it in the patient. The other variant is a heterozygous c.1322T>G substitution in the *solute carrier family 39 member 14* (*SLC39A14* or *ZIP14*) gene (Fig 1B). This variant co-segregates with the disease in the complete family and was not found in 100 control individuals with the same ethnic background and is not present in sequence databases, including dbSNP, 1000 Genomes Project and ExAc databases. Eighteen exons from the linkage region remained partially or completely uncovered by WES and were all checked with Sanger sequencing, but no additional pathogenic variants were identified. Our results therefore indicate that the c.1322T>G variant found in *ZIP14* is the only coding variant in the 8p21 region previously linked to HCI, confirming its disease causality.

### Characterization of the p.L441R variant in *ZIP14*

The human *SLC39A14* gene has four protein coding isoforms according to the National Center for Biotechnology Information (NCBI), all consisting of nine exons. The heterozygous c.1322T>G substitution in exon 8 of *ZIP14* affects all isoforms of the gene and results in a p.L441R substitution (Fig 1C), altering a highly conserved amino acid. Accordingly, this missense mutation has a Combined Annotation Dependent Depletion (CADD) score of 29.4, indicating it belongs to the top 0.11% most deleterious substitutions that can occur in the human genome [5]. As a zinc (Zn) transporter, ZIP14 has six or eight transmembrane domains, depending on the literature or prediction program used (TMHMM, MEMSAT, PRED-TMR, HMMTOP) [6, 7]. Nevertheless, the p.L441R mutation is always located at the end of the second-to-last transmembrane domain of ZIP14. All transmembrane prediction programs predict the variant to cause one or more shifts in a preceding, the affected or the following transmembrane domain, due to the replacement of a hydrophobic leucine by a hydrophilic arginine.

### The p.L441R mutation affects localization and function of *ZIP14 in vitro*

**Fig 2:**
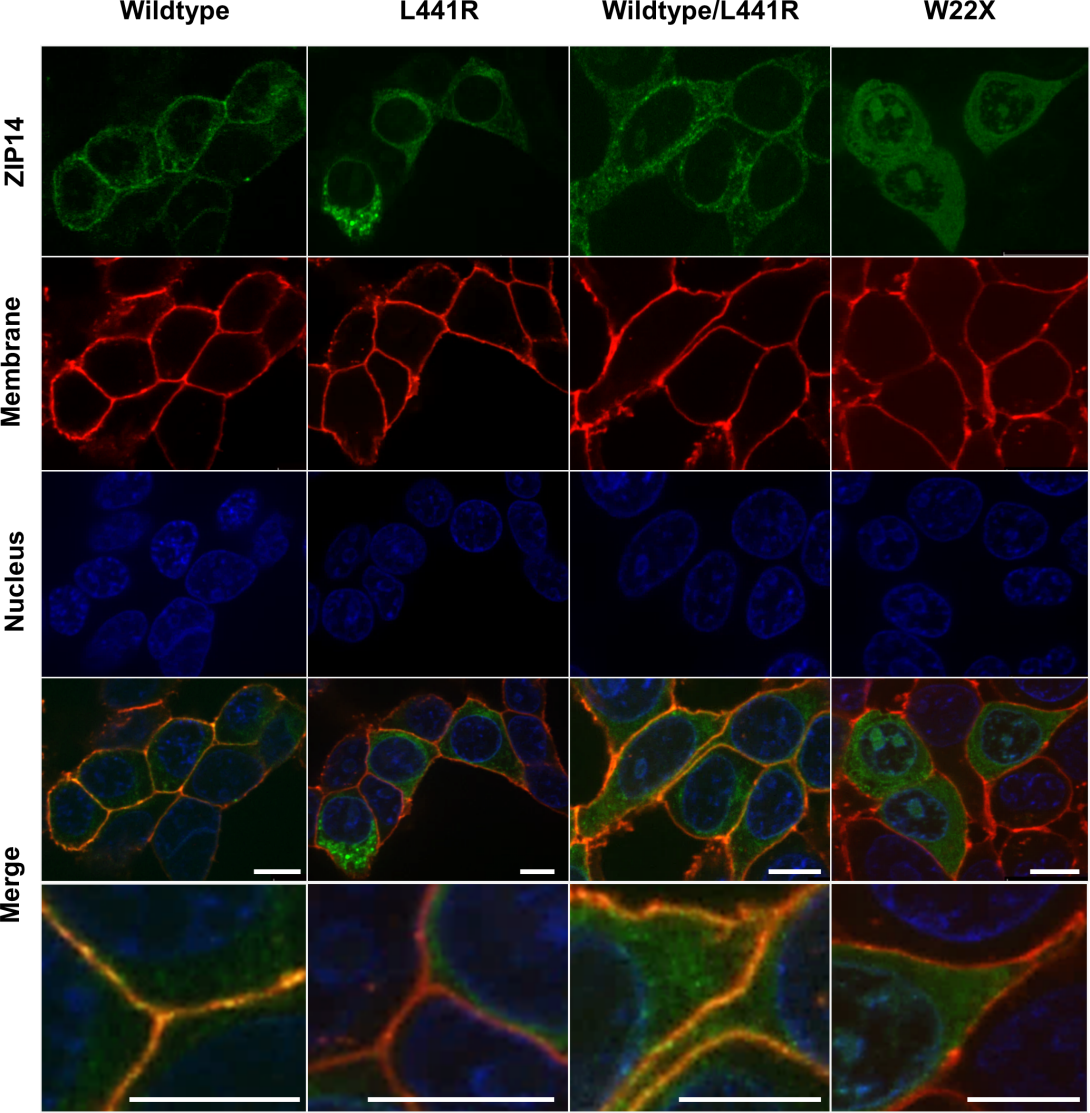
The p.L441R mutation in ZIP14 results in trafficking defects towards the plasma membrane of HEK293T cells. WT ZIP14 is located on the plasma membrane and in the cytoplasm, whereas L441R ZIP14 is no longer present on the plasma membrane, but clusters in the cytoplasm. A heterozygous model (WT/L441R ZIP14) shows increased expression in the cytoplasm (compared to WT) and some co-localization on the plasma membrane. W22X ZIP14 shows expression in the cytoplasm as well as in the nucleus. Scale bars, 10μm.

To evaluate the subcellular localization of mutant (L441R) ZIP14, HEK293T cells were transfected with wildtype (WT), L441R or truncated (W22X) ZIP14-GFP constructs and visualized with confocal microscopy (Fig 2). WT ZIP14 is located on the plasma membrane and in the cytoplasm, as previously reported [8–12]. In contrast herewith, L441R ZIP14 is not present on the plasma membrane, but appears to be trapped in the cytoplasm. Further investigation of the cytoplasmic localization of L441R ZIP14 with markers for the Golgi apparatus and for early and late endosomes demonstrated no difference in the intracellular localization of WT and L441R ZIP14 (S1 Fig). A heterozygous model (WT/L441R ZIP14) clearly shows increased expression in the cytoplasm (compared to WT) and some co-localization on the plasma membrane. W22X ZIP14 shows expression in the cytoplasm as well as in the nucleus. Moreover, there is a difference in cytoplasmic distribution of the different ZIP14 forms, i.e. both WT and L441R ZIP14 appear to be clustered in similar vesicular-shaped structures, whereas W22X ZIP14 is uniformly spread across the cytoplasm (Fig 2).

^65^Zn uptake and Zn accumulation studies were performed to assess the basic functionality of p.L441R ZIP14 as a transporter of Zn (and other metals) (Fig 1D and 1E). ^65^Zn uptake experiments revealed that overexpressing WT ZIP14 significantly (*p*<0.001) increases ^65^Zn uptake by 4-fold when compared to cells transfected with empty vector. On the contrary, L441R and W22X ZIP14 showed no sign of ^65^Zn uptake from the extracellular space into the cell (Fig 1D). This was no surprise, as L441R and W22X ZIP14 were no longer detected on the plasma membrane of the cells. FluoZin3-AM measures the accumulation of labile Zn in the cell. Results show that there is a significant (*p*<0.05) increase in Zn accumulation in cells overexpressing WT ZIP14. Overexpression of L441R ZIP14 also results in a significant (*p*<0.001) increase in intracellular Zn accumulation, which is greater than for WT ZIP14, indicating that labile Zn is trapped in cells with L441R ZIP14 (Fig 1E). Altogether, L441R ZIP14 no longer reaches the plasma membrane, but still resides on the same cytoplasmic structures as WT ZIP14 from where it causes an entrapment of labile Zn.

### *ZIP14* is expressed in osteoblasts and osteoclasts

**Fig 3:**
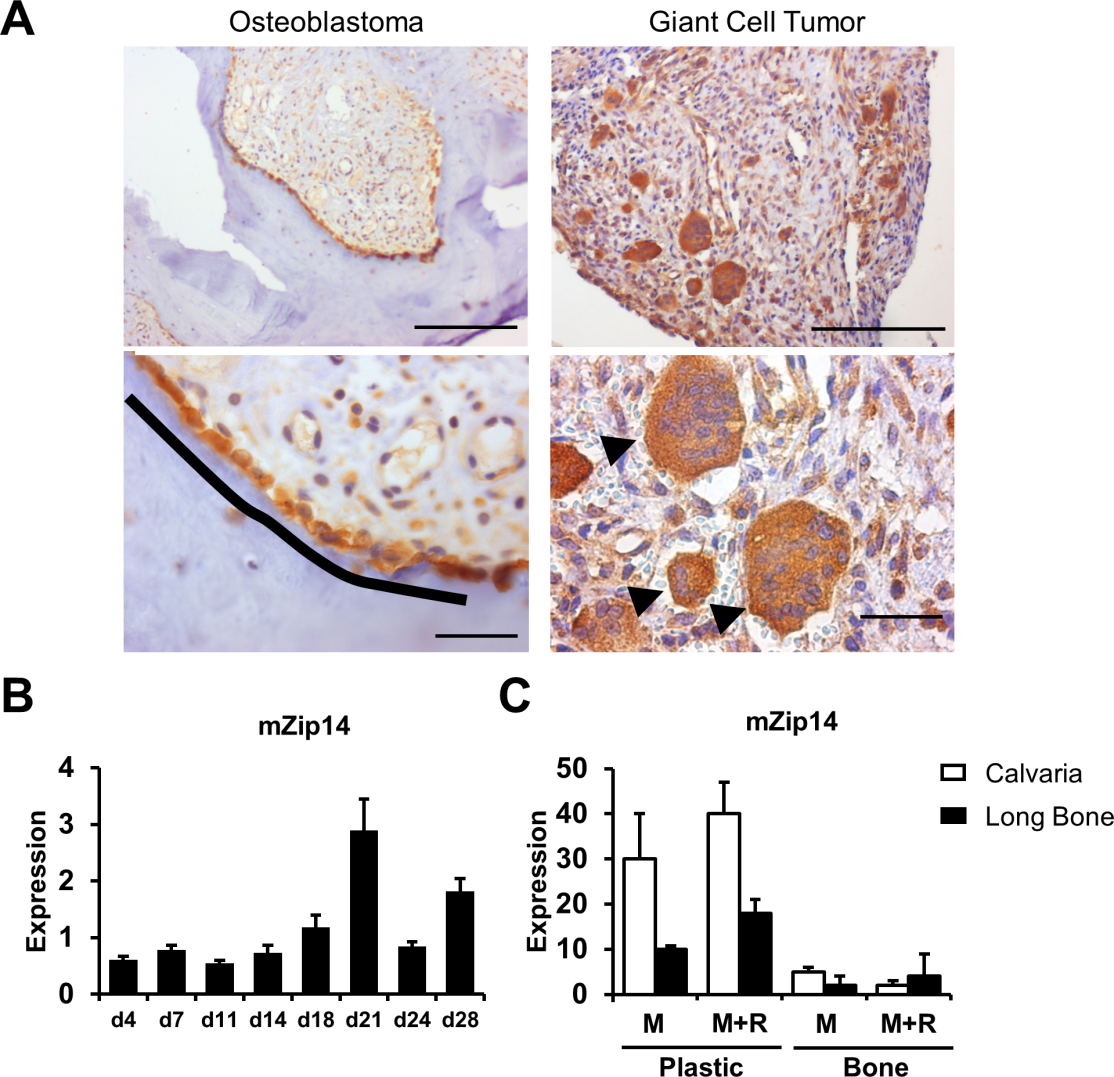
*ZIP14*/*mZip14 is* expressed by both osteoblasts and osteoclasts. **(A)** Immunohistochemistry of osteoblastoma and giant cell tumor tissue depicts *ZIP14* expression in osteoblasts (black line), in giant osteoclast-like cells (arrowheads) and not in osteocytes. Scale bars upper figures, 500μm; scale bars lower figures, 100μm **(B)** Expression of murine *Zip14* (*mZip14*) during differentiation of KS483 cells to mature mineralizing osteoblasts, indicating stable expression of *mZip14* during proliferation (day 4–7) and maturation (day 11–14) of osteoblast differentiation and rising expression during mineralization (day 18–21). **(C)** *In vitro* generated osteoclasts from murine calvariae and long bones were cultured on plastic or cortical bone slices, supplemented with M-CSF (M) or M-CSF + RANKL (M+R). qPCR analysis indicates expression of *mZip14* in osteoclasts derived from calvariae and long bones.

*ZIP14* was reported to be expressed in many tissues with increased expression in the liver, pancreas, thyroid gland, heart and intestine, and a low expression in the brain [6]. Information on the expression of *ZIP14* in skeletal cell types (osteoblasts, osteoclasts, osteocytes) has not been reported in the literature. We therefore performed immunohistochemistry on sections of giant cell tumor and osteoblastoma tissue, bone tumors known to be enriched with osteoclast-like giant cells and osteoblasts, respectively. Here, expression of *ZIP14* was detected in osteoblasts of osteoblastoma tissue and giant cells from giant cell tumor tissue (Fig 3A). *ZIP14* was not expressed in osteocytes of osteoblastoma or giant cell tumor tissue. Moreover, quantitative real-time PCR (qPCR) was performed on KS483 cells, murine mesenchymal stem cells, to assess expression level of murine *Zip14* (*mZip14*) during the different phases of osteoblast differentiation to a mature mineralizing osteoblast. Our results, depicted in Fig 3B, indicate that expression of *mZip14* is stable during proliferation (first week) and maturation (second week) of osteoblast differentiation and rises during the mineralization phase (day 18–21). Lastly, Zip14 expression was checked with qPCR in murine osteoclasts derived from calvaria and long bones. Here we also detected expression of *mZip14* in both osteoclastic cell populations, but in osteoclasts derived from the calvaria we found an average 2-fold greater expression of *mZip14* in osteoclasts (Fig 3C).

### Hyperostosis Cranialis Interna exclusively affects the inner cortex of the skull

**Fig 4:**
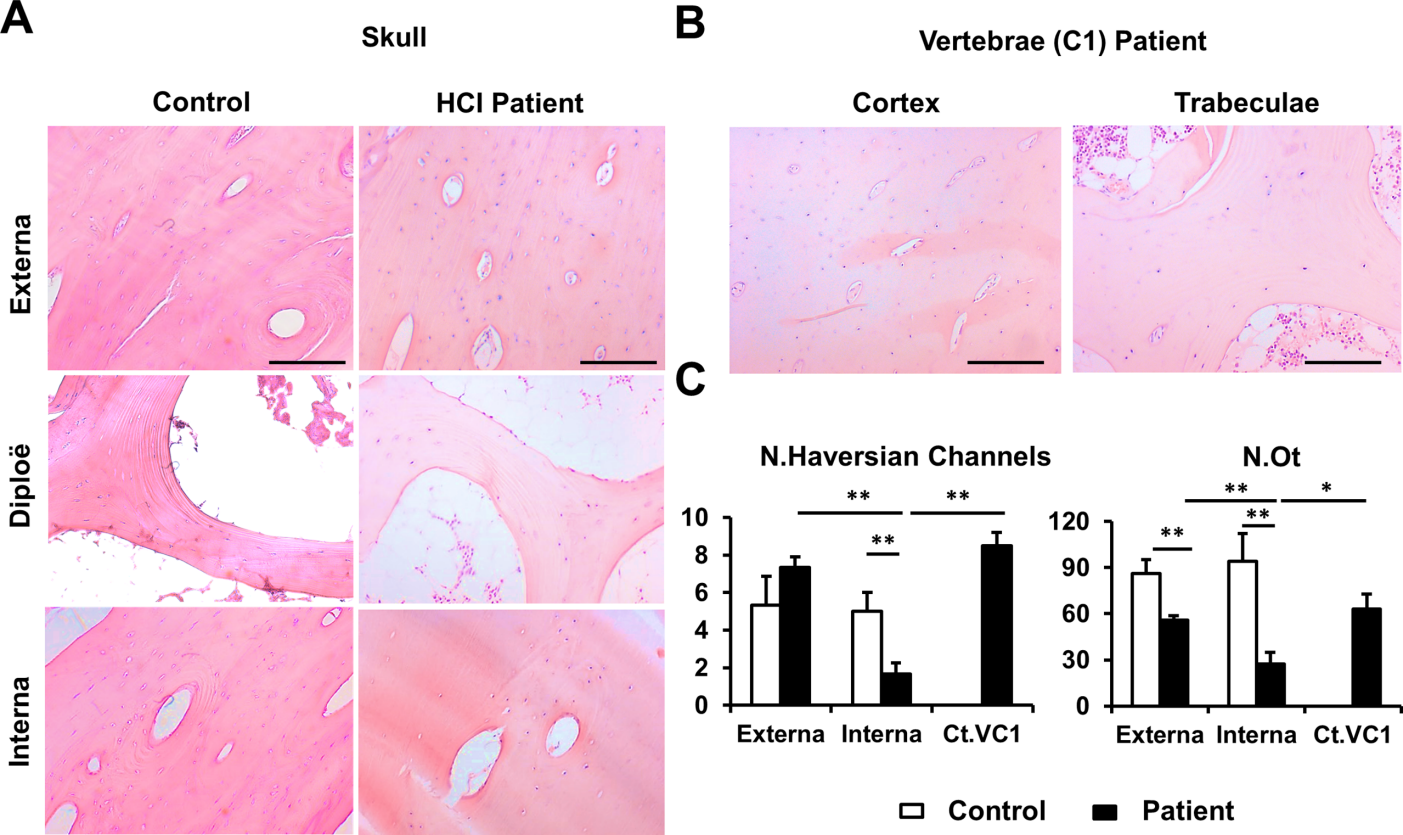
Hyperostosis Cranialis Interna exclusively affects the internal cortex of the skull. **(A)** Microscopic images of the external cortex (externa), diploë and internal cortex (interna) of a patient and control skull biopsy specimen. The structure of the interna and externa of the control are both comparable to the patient externa, whereas the patient interna is clearly affected. The latter demonstrates with a dense amount of well-organized bone and less osteocytes that are grouped around the Haversian channels. **(B)** The cortex and trabeculae of the first cervical (C1) vertebra of the patient show no micro-structural differences with the externa or diploë of the skull of this same patient. **(C)** Quantitative histological analysis of the number of Haversian channels (N.Haversian channels) and number of osteocytes (N.Ot) in the patient and control internae and externae and the cortex of the first cervical vertebra (Ct.VC1) of the patient. In the patient, the number of Haversian channels and osteocytes were both significantly lower in the interna, compared to the externa and the cortex of the cervical vertebra. When comparing the internae of patient and control, both parameters were significantly lower in the patient interna. The externae of patient and control only differ in the number of Haversian channels. *:*p*<0.05; **:*p*<0.01 by 2-tailed Student’s t-test. Scale bars, 500μm.

A skull and first cervical vertebra biopsy specimen were obtained from a patient with HCI as well as a skull biopsy from a control during a neurosurgical intervention. All fragments were embedded in paraffin, sectioned and stained with H&E to examine the micro-structure of the internal cortex (interna), diploë and external cortex (externa) of the skull (Fig 4A). First, in the control sample we did not find significant microscopic differences between the interna and externa (Fig 4C), but in the patient samples the interna is severely affected. The number of Haversian channels and osteocytes is significantly lower in the patient interna, compared to the externa and the cortex of the cervical vertebra of the patient (Fig 4C). When we compare the externa of the patient with that of the control, the number of osteocytes was significantly lower (*p* = 0.0054) in the patient (Fig 4C), although osteocyte distribution is comparable (Fig 4A). Comparing the patient and controle internae, however, demonstrates that the patient interna is wider and characterized by a great and dense amount of well-organized bone, suggesting an increased bone formation or decreased bone resorption. Moreover, the number of Haversian channels (*p* = 0.0075) and the number of osteocytes (*p* = 0.0042) are significantly lower in the patient interna, compared to interna of the control. Remarkably, the osteocytes in the patient interna appear grouped around the Haversian channels. Some osteocyte lacunae, especially further away from the Haversian channels, appear empty, suggesting osteocyte apoptosis. This was not seen in the patient externa or vertebral tissue or in the skull of the control.

### *Zip14*^*-/-*^ mice do not have a calvarial phenotype

**Fig 5:**
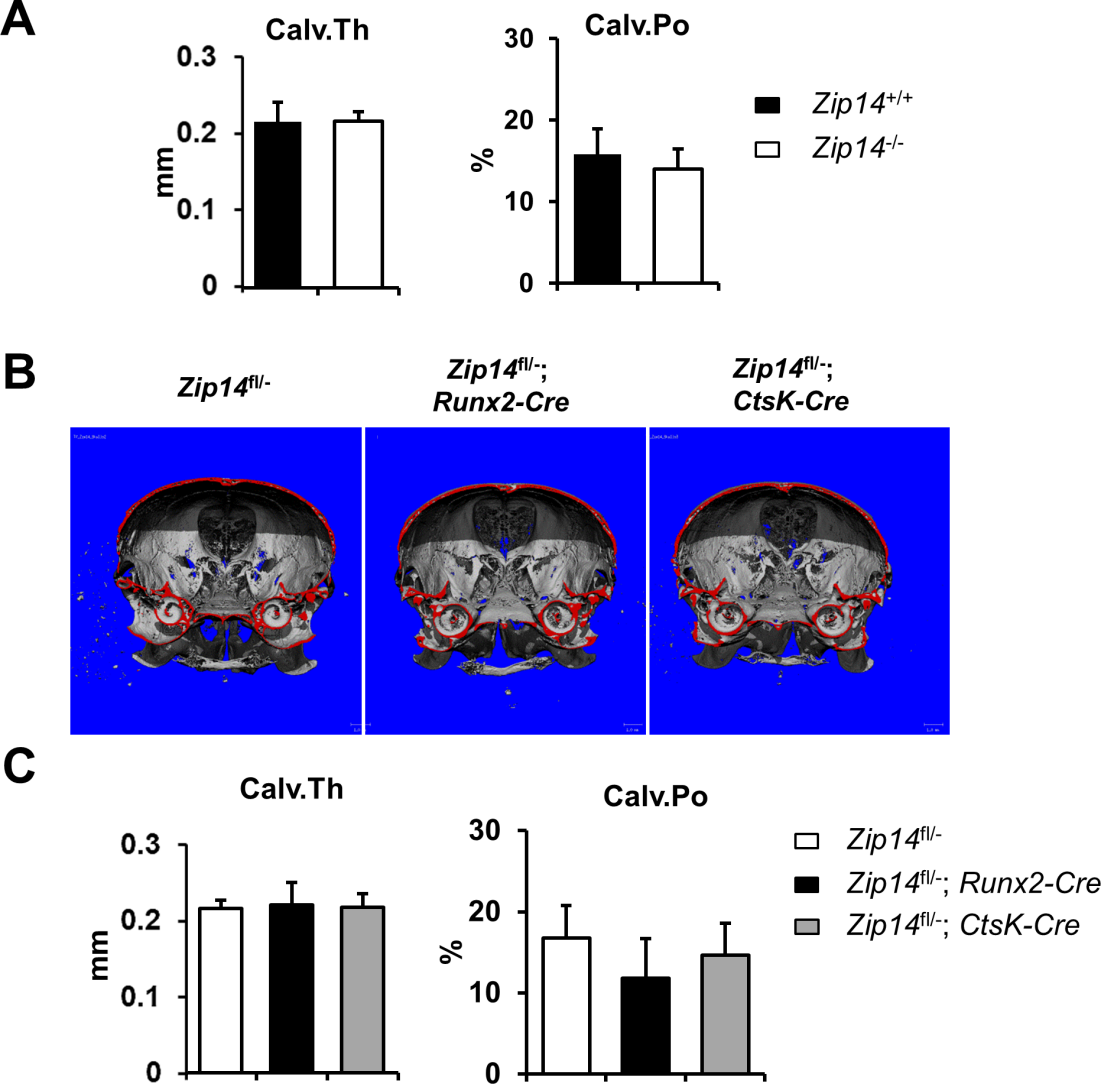
*Zip14*^*-/-*^ mice and conditional Zip14^L438R^ knock-in mice do not have a calvarial phenotype. **(A)** Calvarial thickness (Calv.Th) and porosity (Calv.Po) measured at the calvariae of *Zip14*^*-/-*^ and *Zip14*^*+/+*^ mice shows no significant (*p*<0.05) differences. **(B)** 3D reconstruction of calvariae of *Zip14*^*fl/-*^, *Zip14*^*fl/-*^*; Runx2-Cre* and *Zip14*^*fl/-*^*; CtsK-Cre* mice. **(C)** Calvarial porosity appears lower in *Zip14*^*fl/-*^*; Runx2-Cre* mice, but no significant (*p*<0.05) differences were observed in *Zip14*^*fl/-*^*; Runx2-Cre* and *Zip14*^*fl/-*^*; CtsK-Cre* mice. N = 6 animals/genotype.

*Zip14*^*-/-*^ mice were previously generated at the University of Florida, USA [13]. These *Zip14*^*-/-*^ mice show dwarfism and general osteoporosis of the appendicular skeleton and vertebral column, with a decrease in trabecular bone volume, but normal cortical bone [14]. As no information was available on the calvarial phenotype of these mice, we performed μCT analysis on calvaria of *Zip14*^*+/+*^ and *Zip14*^*-/-*^ mice but found no significant differences in calvarial thickness (Calv.Th) or porosity (Calv.Po) (Fig 5A).

### Ubiquitous expression of Zip14^L438R^ *in vivo* results in perinatal lethality

An *in vivo* model to study the effect of ZIP14^L441R^ was generated by creating a floxed mutant Zip14 (Zip14^flox^) mouse model to express Zip14^L438R^ ubiquitously (Sox2-Cre) or in specific cell types, i.e. osteoblasts (Runx2-Cre) and osteoclasts (CtsK-Cre). Breeding Zip14^flox/flox^ mice with Sox2-Cre mice demonstrated that ubiquitous expression of mutant Zip14 results in perinatal lethality. We therefore focused on mice with conditional expression of Zip14^L438R^. In total, 6-month old *Zip14*^*fl/-*^ controls (n = 6), *Zip14*^*fl/-*^; *Runx2-Cre* (osteoblast-specific knock-ins, Zip14^L438R^ Ob-KI, n = 6) and *Zip14*^*fl/-*^; *CtsK-Cre* (osteoclast-specific knock-ins, Zip14^L438R^ Oc-KI, n = 6) were collected for skeletal phenotyping. No gender-specific differences were observed, so the results presented in this article are solely these from the skeletal analysis of male mice. Skeletal phenotyping results of 6-month old female *Zip14*^*fl/-*^ controls (n = 3), *Zip14*^*fl/-*^; *Runx2-Cre* (n = 3) and *Zip14*^*fl/-*^; *CtsK-Cre* (n = 3) can be found in S2–S4 Figs.

### Osteoblast expression of Zip14^L438R^ differentially modulates cortical and trabecular bone *in vivo*

**Fig 6:**
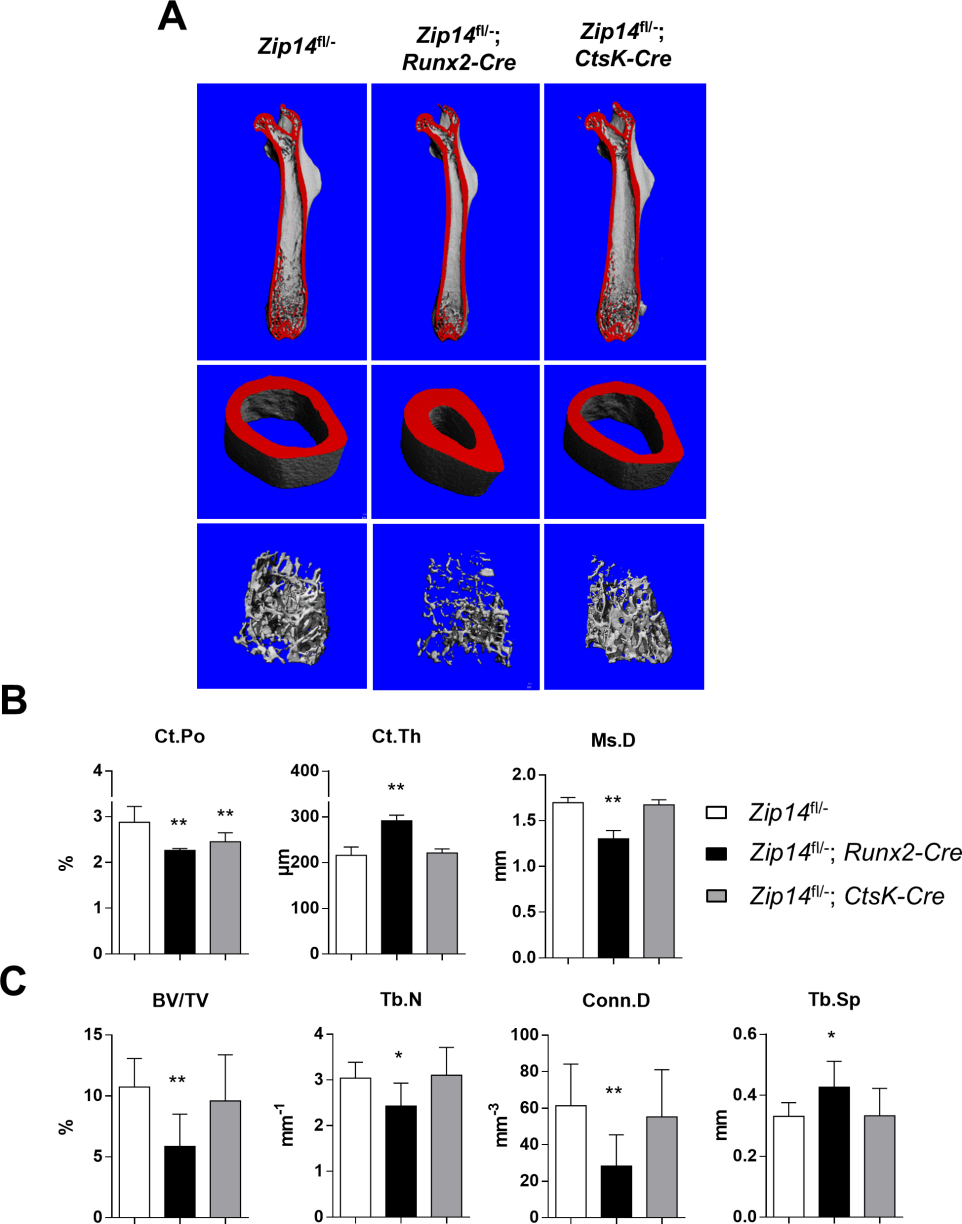
*Zip14*^*fl/-*^*; Runx2-Cre* mice have a severe cortical and trabecular long bone phenotype, whereas *Zip14*^*fl/-*^*; CtsK-Cre* mice do not. **(A)** 3D reconstruction of whole femora (top) and a vertical section of cortical (middle) and trabecular bone (bottom) of *Zip14*^*fl/-*^ controls, *Zip14*^*fl/-*^*; Runx2-Cre* and *Zip14*^*fl/-*^*; CtsK-Cre* mice. Femora of *Zip14*^*fl/-*^*; Runx2-Cre* mice show an increased cortical thickness and decreased midshaft diameter along with a decreased trabecular bone mass. **(B) μ**CT analysis of cortical (Ct) bone parameters confirms a significantly increased cortical thickness (Ct.Th) and decreased midshaft diameter (Ms.D) of *Zip14*^*fl/-*^*; Runx2-Cre* mice. Both *Zip14*^*fl/-*^*; Runx2-Cre* and *Zip14*^*fl/-*^*; CtsK-Cre* mice have a decreased cortical porosity (Ct.Po). **(C) μ**CT analysis of trabecular (Tb) bone parameters confirms a significantly decreased trabecular bone volume (BV/TV), number (Tb.N), connecting density (Conn.D) and increased separation (Tb.Sp) in *Zip14*^*fl/-*^*; Runx2-Cre* mice. N = 6 animals/genotype; *: *p*<0.05; **: *p*<0.025 by 2-tailed Student’s t-test (compared to *Zip14*^*fl/-*^ mice).

μCT analysis of the calvaria and femora was performed to unravel structural differences of Zip14^L438R^ Ob-KI mice versus *Zip14*^*fl/-*^ controls. Although calvarial porosity appears lower in these mice there were no significant differences in calvarial parameters (Fig 5B). In contrast herewith, μCT analysis of the femora showed a severe skeletal phenotype versus controls (Fig 6). Compared to *Zip14*^*fl/-*^ controls, the Zip14^L438R^ Ob-KI mice had a significant increased cortical thickness (Ct.Th, *p* = 6.0E-6) with a decreased cortical porosity (Ct.Po, *p* = 0.0014) and a significantly smaller midshaft diameter (Ms.D, *p* = 4.1E-6) (Fig 6A and 6B). Furthermore, Zip14^L438R^ Ob-KI mice have a significantly decreased trabecular bone volume (BV/TV, *p* = 0.0071), number (Tb.N, *p* = 0.033) and connecting density (Conn.D, *p* = 0.018) with an increased trabecular separation (Tb.S, *p* = 0.035) (Fig 6C).

**Fig 7:**
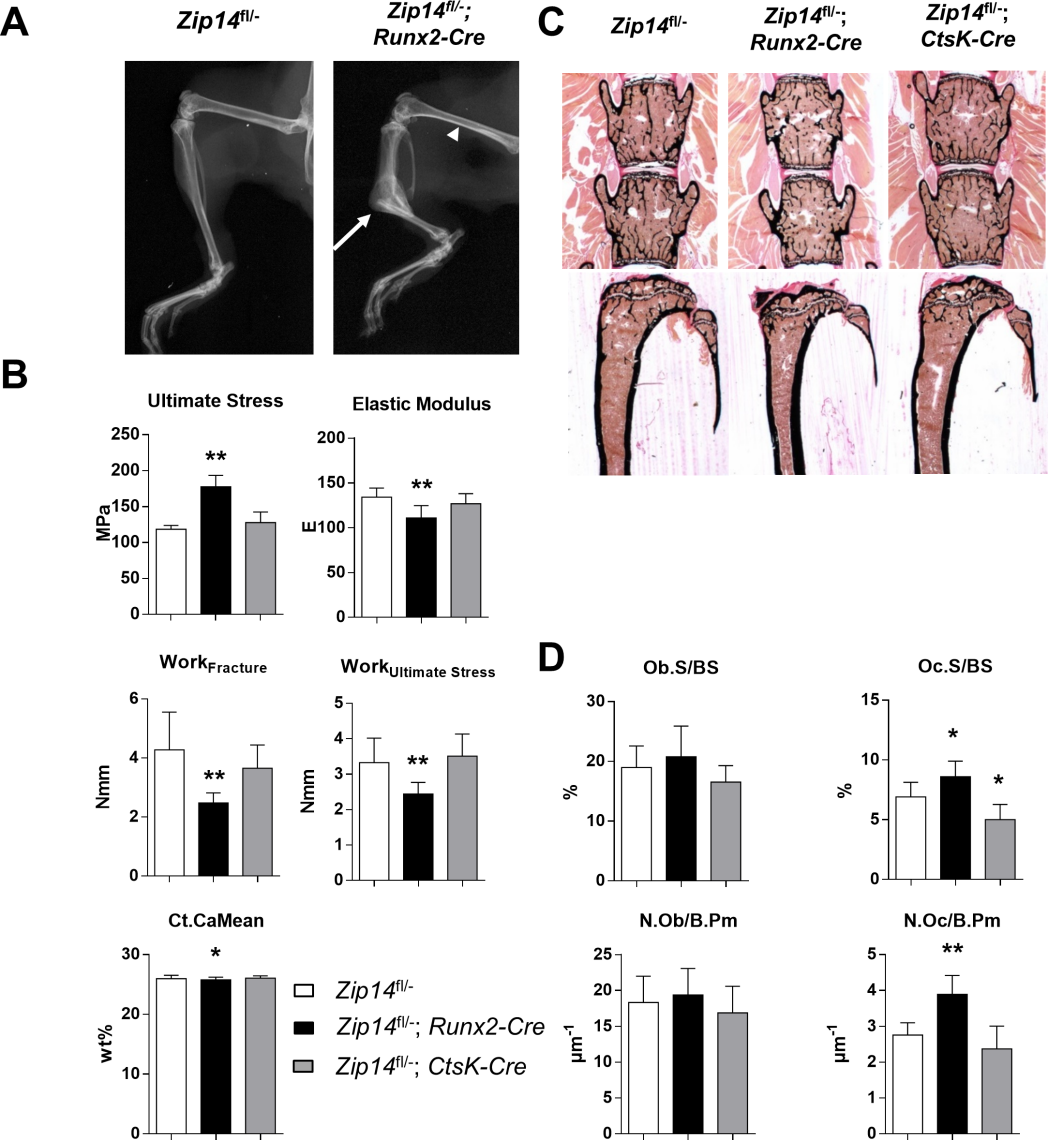
Long bones of *Zip14*^*fl/-*^*; Runx2-Cre* mice are more fragile and have a significant higher number of osteoclasts. **(A)** X-rays of femora and tibiae of a *Zip14*^*fl/-*^ and *Zip14*^*fl/-*^*; Runx2-Cre* mouse. The latter demonstrates with a tibial fracture (arrow), whereas narrowing of the femoral midshaft can be observed as well (arrowhead). **(B)** Three-point bending analysis indicates that femora of *Zip14*^*fl/-*^*; Runx2-Cre* mice tolerate higher stress levels and are more elastic. Work-to-fracture is also significantly reduced and qBEI analysis indicates significant lower cortical mineralization (Ct.CaMean) of femora of these mice, compared to controls. **(C)** Representative undecalcified spine (upper row) and tibia sections (bottom row) from *Zip14*^*fl/-*^, *Zip14*^*fl/-*^*; Runx2-Cre* and *Zip14*^*fl/-*^*; CtsK-Cre* mice stained with von Kossa/van Gieson. Vertebrae of *Zip14*^*fl/-*^*; Runx2-Cre* mice show less trabecular bone, whereas tibiae of these mice show an increased cortical thickness and decreased midshaft diameter compared to *Zip14*^*fl/-*^ controls. **(D)** Quantification of the bone surface covered by osteoblasts (Ob.S/BS), osteoblast number per bone perimeter (N.Ob/B.Pm), osteoclast surface per bone surface (Oc.S/BS) and osteoclast number per bone perimeter (N.Oc/B.Pm) in the vertebral bodies analyzed using toluidine blue staining. N = 6 animals/genotype; *: *p*<0.05; **: *p*<0.025 by 2-tailed Student’s t-test (compared to *Zip14*^*fl/-*^ mice).

**Fig 8:**
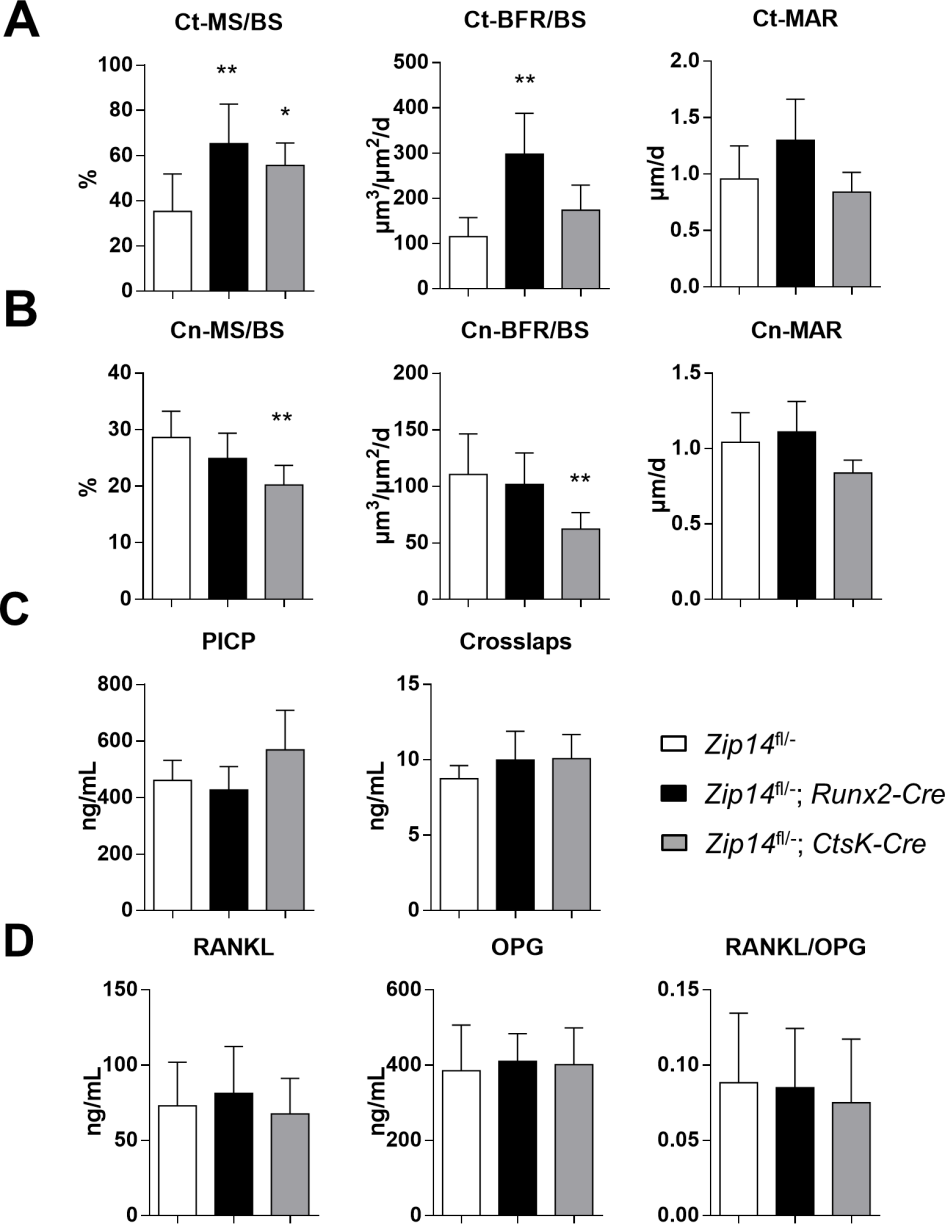
An increased endosteal bone formation underlies the cortical long bone phenotype of *Zip14*^*fl/-*^*; Runx2-Cre* mice. **(A)** Dynamic histomorphometry of the tibial endocortical (Ct) bone surface indicates a significant increase in endosteal mineralizing surface (MS/BS) and bone formation rate (BFR/BS) in *Zip14*^*fl/-*^*; Runx2-Cre*. In *Zip14*^*fl/-*^*; CtsK-Cre* mice a significant increase in MS/BS was detected. **(B)** Dynamic histomorphometry of trabecular (Tb) bone surface of vertebral bodies (L3-L4) indicates a significant lower MS/BS and BFR/BS in *Zip14*^*fl/-*^*; CtsK-Cre* mice. **(C)** Measurement of the bone turnover markers procollagen type-I C-terminal peptide (PICP; bone formation) and collagen type 1 cross-linked C-telopeptide (Crosslaps; bone resorption) in serum of 6-month old control (*Zip14*^*fl/-*^), osteoblast-specific knock-in (*Zip14*^*fl/-*^*; Runx2-Cre*) and osteoclast-specific knock-in (*Zip14*^*fl/-*^*; CtsK-Cre*) mice. **(D)** Quantification of the serum RANKL and OPG levels and the calculated RANKL/OPG ratio. N = 6 animals/genotype; *: *p*<0.05; **: *p*<0.025 by 2-tailed Student’s t-test (compared to *Zip14*^*fl/-*^ mice).

X-ray radiographs of the whole skeletons indicated a fracture with callus in the tibiae of two Zip14^L438R^ Ob-KI mice (arrow, Fig 7A). Moreover, as seen in the μCT analysis of the femora, X-rays also revealed severe narrowing of the femoral midshaft in these mice (arrowhead, Fig 7A). Assessment of the biomechanical properties of the femora with three-point bending tests indicated that they bear significant higher stress levels (*p* = 5.0E-4) but work-to-fracture was 42% percent lower (*p* = 0.0086) than of femora of controls, probably due to the observed changes in cortical thickness and midshaft diameter. The elastic modulus (*p* = 0.013) and work to reach ultimate stress levels (*p* = 0.021) were also significantly lower in femora of these mice, suggesting more elastic femora (Fig 8B). Consequently, qBEI analysis indicated a significantly reduced cortical mineralization (Ct.CaMean, *p* = 0.026), contributing to this increased flexibility. This clearly illustrates that expression of Zip14^L438R^ in osteoblasts results in more fragile and more flexible femora *in vivo*.

Undecalcified sections of lumbar vertebral bodies and tibiae were stained with Von Kossa/Van Gieson staining, as depicted in Fig 7C. Quantification of parameters of structural histomorphometry confirmed the trabecular phenotype observed with μCT analysis (S5 Fig). Sections stained with toluidine blue were analyzed to further investigate the skeletal phenotype on a cellular level. In Zip14^L438R^ Ob-KI mice we observed no significant differences in osteoblast-covered surface (OB.S/BS, *p* = 0.50) or number (OB.N/B.Pm, *p* = 0.63). Surprisingly, the osteoclast-covered surface (OC.S/BS, *p* = 0.043) and number (N.OC/B.Pm, *p* = 0.0012) were significantly increased, compared to *Zip14*^*fl/-*^ controls (Fig 7D).

Double calcein labelling allowed us to investigate the (endosteal and periosteal) cortical and trabecular mineralizing surface (MS/BS), bone formation rate (BFR/BS) and mineral apposition rate (MAR) by fluorescence microscopy. Compared to *Zip14*^*fl/-*^ controls, Zip14^L438R^ Ob-KI mice had an increase in endosteal MS/BS (*p* = 0.012) and even more in BFR/BS (*p* = 0.0012) (Fig 8A), whereas there were no significant differences in periosteal (S6 Fig) or trabecular bone formation parameters (Fig 8B).

Serum was collected prior to euthanasia of the animals for measurement of procollagen I C-terminal propeptide (PICP) and C-terminal telopeptide (CTX Crosslaps) as serum markers for bone formation and resorption, respectively. Zip14^L438R^ Ob-KI mice had similar levels of PICP and CTX, compared to *Zip14*^*fl/-*^ mice (Fig 8C). Serum levels of OPG and RANKL were both slightly higher (not significant) in these mice, resulting in a similar RANKL/OPG ratio as controls (Fig 8D).

**Fig 9:**
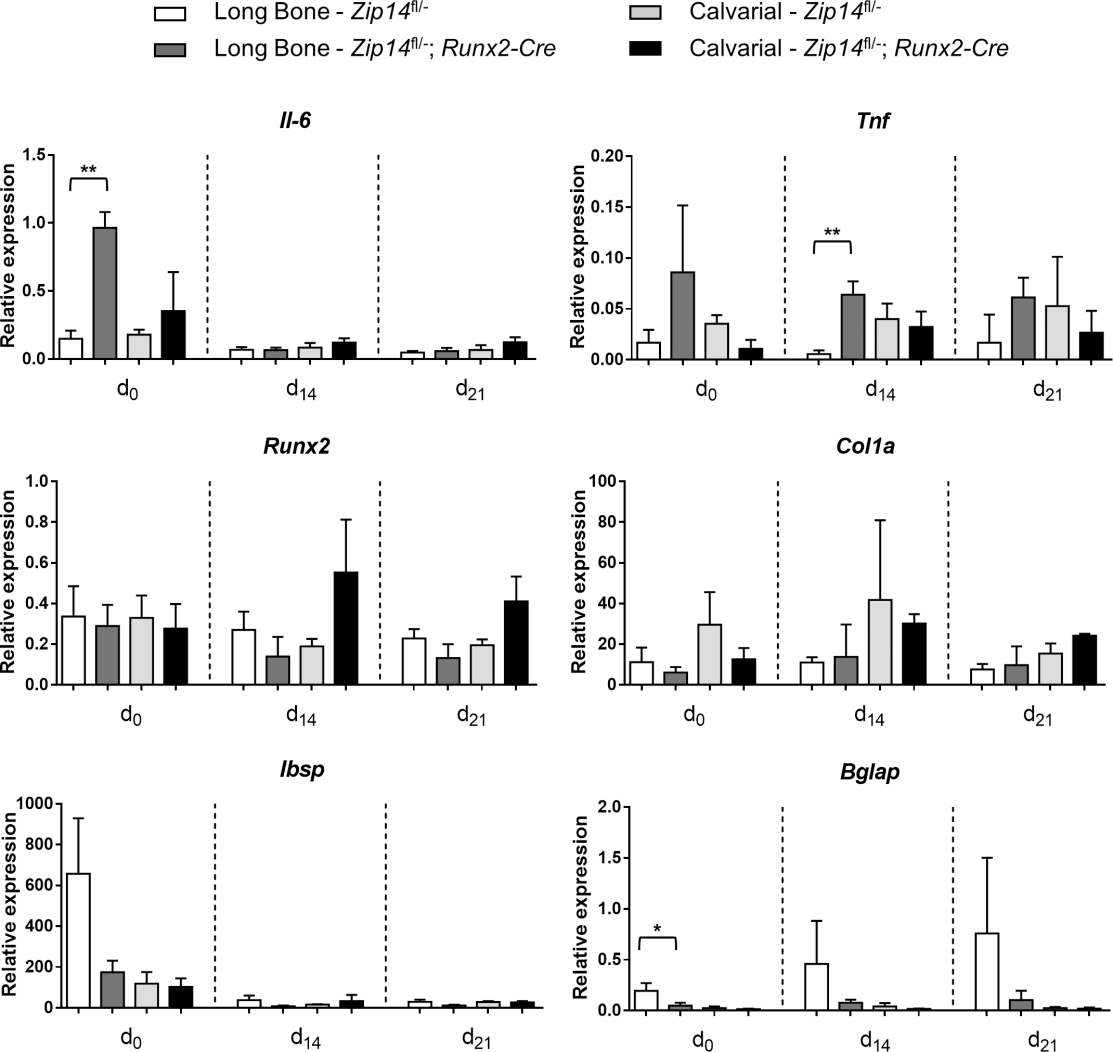
Long bone osteoblasts of *Zip14*^*fl/-*^*; Runx2-Cre* mice show an increased expression of inflammatory cytokines during osteoblast differentiation. qRT-PCR analysis of genes encoding inflammatory cytokines (*Il-6*, *Tnf*) and osteoblast markers (*Runx2*, *Col1a*, *Ibsp*, *Bglap*) was performed in primary osteoblasts derived from the long bones and calvariae of *Zip14*^*fl/-*^ mice and *Zip14*^*fl/-*^*; Runx2-Cre* mice at three time points (d_0_, d_14_, d_21_) during their differentiation. A significant higher expression of *Il-6* and *Tnf* was detected at respectively day 0 and day 14 of differentiating long bone *Zip14*^*fl/-*^*; Runx2-Cre* osteoblasts, compared to long bone *Zip14*^*fl/-*^ osteoblasts. *Bglap* expression was, on the other hand, significantly lower at day 0 of differentiating long bone *Zip14*^*fl/-*^*; Runx2-Cre* osteoblasts. N = 3 animals/genotype; *: *p*<0.05; **: *p*<0.01 by 2-tailed Student’s t-test (compared to *Zip14*^*fl/-*^ mice).

Finally, primary osteoblasts derived from the long bones and calvariae of *Zip14*^*fl/-*^ controls and Zip14^L438R^ Ob-KI mice were isolated and subsequently cultured for 21 days. During this period, RNA was isolated at day 0, day 14 and day 21 of differentiation for qRT-PCR analysis. In calvarial osteoblasts, there was no difference in the expression of osteoblast markers (*Runx2*, *Col1a*, *Ibsp*, *Bglap*) or inflammatory cytokines (*Il-6*, *Tnf*) between controls and Zip14^L438R^ Ob-KI mice (Fig 9). In osteoblasts derived from the long bones of Zip14^L438R^ Ob-KI mice, however, we found a significant higher expression of *Il-6* (day 0) and *Tnf* (day 14); compared to *Zip14*^*fl/-*^ controls. *Bglap* expression was, on the other hand, significantly lower in these osteoblasts at day 0 (Fig 9). As these expression data and the skeletal phenotype were very different in calvaria and long bones of Zip14^L438R^ Ob-KI mice, we additionally verified *Zip14*^*L438R*^ overexpression in calvarial and long bone osteoblasts. Nevertheless, by amplifying and sequencing the region surrounding the c.1535 T>G (p.L438R) mutation in *Zip14*, *Zip14*^*L438R*^ overexpression was confirmed in cDNA from calvarial and long bone osteoblasts of Zip14^L438R^ Ob-KI mice (S8 Fig).

### Osteoclast expression of Zip14^L438R^ has little effect on bone homeostasis *in vivo*

μCT analysis of Zip14^L438R^ Oc-KI mice demonstrated a significantly decreased cortical porosity (*p* = 0.016) compared to *Zip14*^*fl/-*^ controls, whereas trabecular bone was unaffected (Fig 6). Histological analysis of undecalcified Von Kossa/Van Gieson stained spine and tibia sections confirmed trabecular bone mass to be unaffected in these mice (Fig 7C, S2 Fig). Three-point bending tests indicated that biomechanical properties of the femora of these mice were similar to that of *Zip14*^*fl/-*^ controls (Fig 7B). Furthermore, toluidine blue stained sections of the tibiae showed a significant decrease in osteoclast-covered bone surface (*p* = 0.024), whereas osteoclast number (*p* = 0.22) and osteoblast-covered surface (*p* = 0.22) and number (*p* = 0.50) were unaltered (Fig 7D). Regarding dynamic histomorphometry, Zip14^L438R^ Oc-KI mice presented with a significant increase in endosteal mineralizing surface (*p* = 0.039), whereas trabecular MS/BS (*p* = 0.0086) and BFR/BS (*p* = 0.020) were decreased (Fig 8). Finally, serum PICP levels of osteoclast knock-in mice were slightly increased, but did not reach significance, whereas CTX was at the same level as controls (Fig 8C). The RANKL/OPG ratio of osteoclast knock-in mice was somewhat lower, due to a slight decrease in RANKL and increase in OPG. Again, this did not reach significance (Fig 8D).

### ZIP14^L441R^ increases cAMP-CREB and NFAT signaling

**Fig 10:**
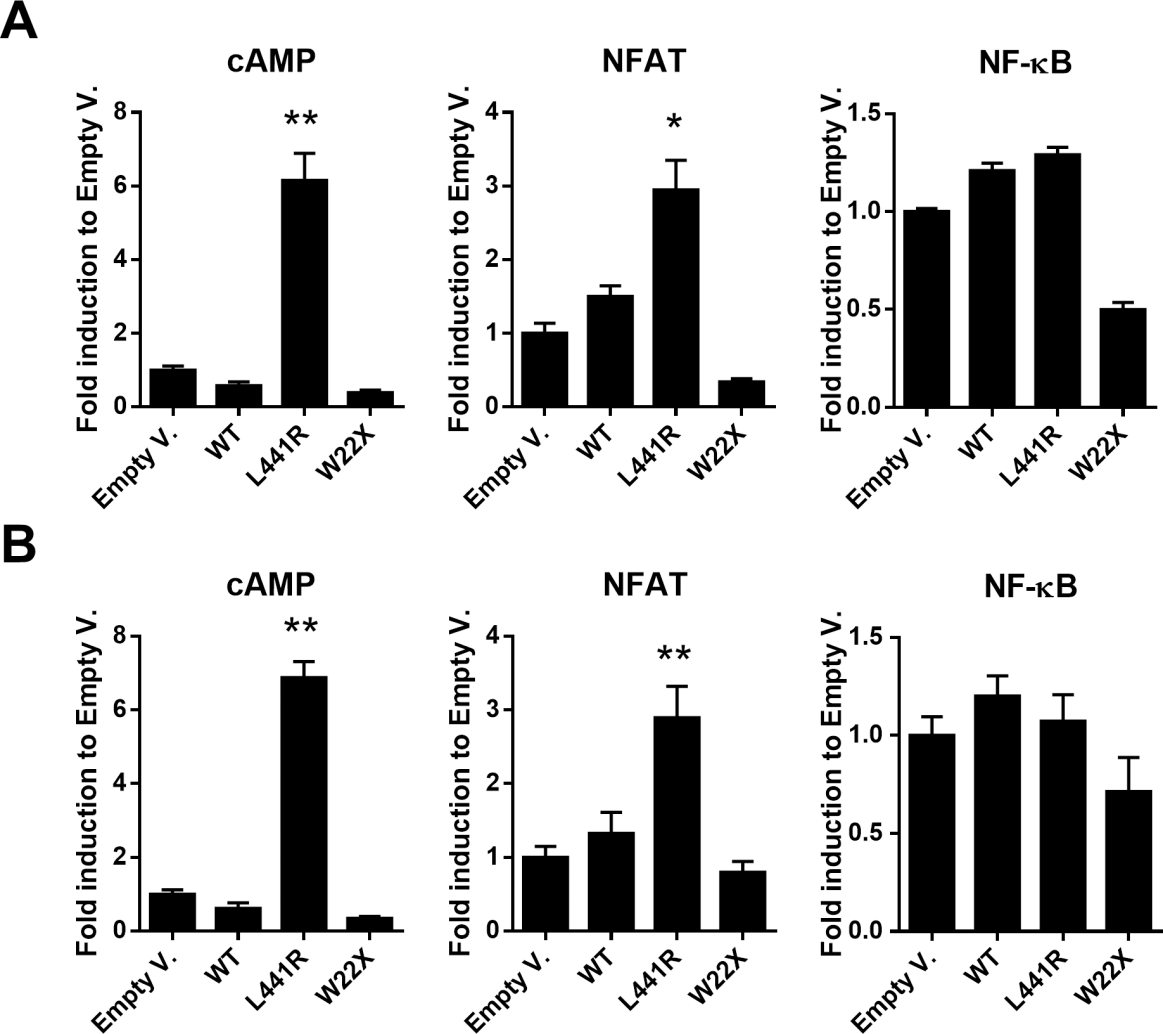
cAMP-CREB and NFAT signaling are significantly increased by L441R ZIP14. Luciferase reporter assays investigating cAMP-CREB activity, NF-κB activity and NFAT activity by wildtype (WT), L441R and truncated (W22X) ZIP14 in **(A)** HEK293T cells and **(B)** Saos-2 cells demonstrate a significant increase in cAMP-CREB and NFAT signaling by L441R ZIP14, compared to WT ZIP14. *: *p*<0.05; **: *p*<0.01 by 2-tailed Student’s t-test.

Zip14 was previously linked to cAMP-CREB signaling [15]. To evaluate the effect of WT and L441R ZIP14 on the cAMP-CREB signaling activity, a luciferase reporter assay with a cAMP-responsive luciferase construct was applied. Here, overexpression of WT ZIP14 in HEK293T caused a decrease in cAMP-CREB signaling, whereas overexpression of L441R ZIP14 resulted in a significant (*p* = 0.004) 5-fold increase in activity (Fig 10A). Next to cAMP-CREB signaling, ZIP14 has been associated with immune response and inflammation in the literature. We therefore checked both NF-κB and NFAT signaling activity, due to their importance in bone cells and their association with inflammatory processes. No significant difference in NF-κB signaling was observed between WT and L441R ZIP14, but NFAT signaling by L441R ZIP14 was significantly increased (*p* = 0.031) compared to WT ZIP14 in HEK293T cells (Fig 10). All luciferase reporter assays were also performed in Saos-2 cells, i.e. osteoblast-like cells, with similar results (Fig 10B).

## Discussion

Hyperostosis Cranialis Interna (HCI, OMIM 144755) was described in a Dutch family as a bone disorder that solely affects the calvaria and skull base through intracranial hyperostosis and osteosclerosis [1, 2]. We performed a whole genome linkage analysis in the past and mapped the disorder to a region on chromosome 8 (8p21) [4]. In this study we additionally performed WES on one HCI patient which led to the identification of a heterozygous c.1322T>G (p.L441R) substitution in the *SLC39A14* gene that co-segregates with the disorder. *SLC39A14* encodes a Zn transporter that belongs to the SLC39A or Zrt-, Irt-related protein (ZIP) family and is therefore often referred to as ZIP14. ZIP transporters invariably function by replenishing cytosolic Zn from the extracellular space and the lumen of intracellular compartments (influx) [16].

ZIP14 has previously been localized to the plasma membrane and in the cytosol, in early and late endosomes [8–12]. From here, ZIP14 mainly mobilizes Zn, but transport of other divalent cations (iron, manganese, cadmium) into the cytosol is also described [17, 18]. We demonstrate that ZIP14^L441R^ is still localized in the early and late endosomes, but loses its presence on the plasma membrane, implying trafficking defects of ZIP14^L441R^ *in vitro*. It is subsequently possible that ZIP14^L441R^ is retained in the endosomes. Of note, patients with HCI have a heterozygous p.L441R substitution, indicating that fifty percent of ZIP14 is wildtype and reaches the plasma membrane (and early/late endosomes), whereas the other fifty percent will reasonably be trapped onto the endosomes. Consistent with the changes in localization, ZIP14^L441R^ was not able to transport Zn from the extracellular space into the cell. Accumulation of labile Zn in the cell, however, was increased by ZIP14^L441R^, indicating an aberrant cellular Zn homeostasis. It is essential to note that the cellular localization of labile Zn excess is currently unknown and depends on transport capacity of ZIP14^L441R^. This is highly relevant as Zn generally plays a vital role in cells as it is estimated that about 10% of the human genome encodes proteins with Zn-binding sites. More than half of those are thought to be transcription factors and enzymes, distributed across the different cellular compartments. Local alterations in Zn homeostasis can therefore have significant effects on the functionality of corresponding Zn-dependent proteins and of cells, which could thus be the basis of the pathogenesis of HCI [16, 19]. Similarly, mutations in *SLC39A4* (*ZIP4*) and *SLC39A13* (*ZIP13*) have been linked to Zn deficiency and/or accumulation in specific cellular compartments resulting in acrodermatitis enteropathica and spondylocheiro dysplastic Ehlers-Danlos syndrome, respectively [20–23].

Next to aberrations in Zn homeostasis, it is important to note that mutations in *ZIP14* can affect manganese (Mn), cadmium and iron homeostasis as well. Recently, homozygous missense, nonsense and frameshift mutations in *ZIP14* were identified in patients with childhood-onset parkinsonism-dystonia, due to defects in Mn homeostasis [12]. These mutations were all part of transmembrane domains that are not predicted to form a pore (according to MemSatSVM), where our mutation is part of. The subcellular localization of all ZIP14 mutants in the study by Tuschl *et al*. were similar to that of wildtype ZIP14, Mn uptake was reduced and specifically accumulated in the brain of a mutant zebrafish model [12]. For our study, we focused on Zn as it is more relevant in skeletal homeostasis [16, 19, 24]. Zn is described to have a stimulatory role on osteoblastic bone formation and mineralization and an inhibitory effect on osteoclastic bone resorption [24, 25] and we demonstrated expression of *ZIP14* in osteoclasts and osteoblasts. Effects of ZIP14^L441R^ on skeletal homeostasis were therefore investigated in conditional knock-in mice with expression of Zip14^L438R^ in osteoblasts or osteoclasts. First, femoral length (growth) was similar for all mice (S8 Fig). This is relevant since Zn deficiency is generally associated with growth retardation (and other symptoms) [16, 19] and *Zip14*^*-/-*^ mice exhibit such phenotype marked by growth retardation and dwarfism [15]. As the role of Zip14 in growth was however attributed to its effects on the hypertrophy of chondrocytes, this could explain the normal growth in our osteoblast or osteoclast knock-in mice. Nevertheless, skeletal growth or height is not affected in patients with HCI as well. Since patients with HCI carry a heterozygous p.L441R mutation and *Zip14*^*+/-*^ mice are phenotypically normal [15], it could be that the wildtype allele fulfills a compensatory role and that growth defects in *Zip14*^*-/-*^ mice are due to a general state of Zn deficiency. Moreover, it was documented that ZIP14 has roles in adipose tissue and glucose utilization that can influence growth of *Zip14*^*-/-*^ mice as well [11, 26].

Knowing the long bones were affected by Zip14^L438R^ in our conditional knock-in mice, we were surprised to see no calvarial phenotype as this is truly opposite of what we see in HCI patients. One aspect to be discussed here is the difference in expression of human *ZIP14*^*L441R*^ and murine *Zip14*^*L438R*^. In HCI patients, endogenous *ZIP14* is expressed in its own spatiotemporal manner, whereas *Zip14*^*L438R*^ expression is driven by the Runx2 and Cathepsin K promoter in our conditional knock-in mice. Nevertheless, *Cre* expression was reported in long bones and calvariae of both Cre-models used in this study [27, 28] and overexpression of *Zip14*^*L438R*^ was confirmed in calvarial and long bone osteoblasts derived from Zip14^L438R^ Ob-KI mice (S8 Fig). Still, we analyzed the calvarial phenotype of *Zip14*^*-/-*^ and *Zip14*^*+/+*^ mice and found that loss of endogenous Zip14 did not affect the calvariae, even though the appendicular skeleton and vertebral column were osteoporotic. This suggests that aberrations in Zn homeostasis by Zip14 do not seem to affect calvariae of mice, even though the rest of the skeleton is affected. Whether this is due to a specific protective mechanism present in murine calvariae but not in humans, remains to be determined.

In contrast to the calvariae, the appendicular skeleton and vertebral column were affected by knock-in of Zip14^L438R^ in osteoblasts and osteoclasts. Generally, knock-in of Zip14^L438R^ in osteoblasts resulted in a severe skeletal phenotype, whereas the skeletal phenotype in osteoclast knock-in mice was milder. Based on these findings, we conclude that osteoblasts are the primary cells through which mutant ZIP14 exerts its effects on bone homeostasis. Nevertheless, a remarkable finding was that both conditional knock-in models had an increased (endo)cortical bone formation rate. Additionally, osteoblast knock-in mice had an increased cortical thickness, where excessive endosteal bone formation even led to narrowing of the bone marrow cavity. Similarly, a study investigating the metabolic activity in the calvariae of HCI patients with ^18^F-fluoride PET/CT depicted the highest rates of ^18^F-fluoride uptake in the hyperostotic regions and more specifically at the endosteal side of the diploe (towards the bone marrow)[29]. Bone overgrowth of the inner calvarial cortex of HCI patients is thus also the result of an increased endosteal bone formation. Therefore, even though the location of the skeletal defect is different, i.e. in the appendicular skeleton and vertebral column *versus* the calvaria, the (endo)cortical phenotype and the underlying cause of this are strikingly similar in Zip14^L438R^ osteoblast knock-in mice and HCI patients.

To further elucidate the *in vivo* effects of Zip14^L438R^ through osteoblasts, we focused on the fact that Zip14^L438R^ has disparate effects on cortical and trabecular bone in Zip14^L438R^ Ob-KI mice. These mice had an increased cortical thickness and narrowed bone marrow cavity along with a decreased trabecular bone volume. According to the literature, only few hormones and pathways have similar effects on the skeleton and these are parathyroid hormone (PTH)/parathyroid-related protein (PTHrP) and estrogen. Of note, Zip14 was previously associated with PTH1R-cAMP-CREB signaling in *Zip14*^*-/-*^ mice [15]. *Pth*^*-/-*^ mice and mice with osteoblast/osteocyte-specific G_s_α deficiency (BGsKO), bearing in mind that PTH mediates its effects through G_s_α signaling, have an increased cortical bone mass, decreased bone marrow cavity and a decreased trabecular bone mass in both models [30, 31]. Albeit more severe, this phenotype has the same differential effects on bone as seen in our Zip14^L438R^ Ob-KI mice. A contrasting skeletal phenotype is also seen in mice with PTH/PTHrP receptor overexpression in the osteoblastic lineage [32]. This suggests that the skeletal phenotype of Zip14^L438R^ Ob-KI mice resembles that of deficient or restrained PTH-signaling in osteoblasts.

Despite the fact that estrogen was not previously associated with Zip14, it exerts opposing actions on bone compared to PTH in osteoblasts and studies show that Zn has actions similar to estrogen on osteoblasts and osteoclasts [25, 33]. Estrogen is generally known to restrain periosteal and stimulate endosteal bone formation during bone modeling and remodeling through osteoblast progenitors [33, 34]. Consequently, postmenopausal sex-steroid deficiency has been associated with an enlargement of the marrow cavity, thinning of the cortex and slight increase in midshaft diameter [35]. Zip14^L438R^ Ob-KI mice, on the contrary, have a smaller midshaft diameter, due to a restricted periosteal bone formation, along with a thicker cortex and narrowed bone marrow cavity, resulting from a stimulated endosteal bone formation. Moreover, estrogen has protective effects on the resorption of both trabecular and cortical bone, but these are exerted by disparate cell types, i.e. by direct effects on osteoclasts and indirect effects on osteoblasts, respectively [33]. A possible explanation for the trabecular phenotype of Zip14^L438R^ Ob-KI mice is that by sole osteoblastic expression of Zip14^L438R^, there is no protective (estrogen-mimicking) effect on the resorption of trabecular bone. Another important hint for a role of estrogen-like signaling by mutant ZIP14 was found in clinical reports on the disease progression of HCI patients. Female patients exhibit sudden aggravation of HCI symptoms during pregnancy, like abrupt loss of smell or hearing, of which they sometimes recovered after pregnancy. Furthermore, female patients are often more severely, albeit not significant, affected by HCI [2]. As mentioned in the introduction, radiological abnormalities associated with HCI are often seen in the first decade of life and a slow progression of the disease can be seen until the age of 40 [2, 3]. Altogether, these stages in life share critical changes in estrogen levels, i.e. estrogen gain associated with puberty and pregnancy and estrogen loss associated with aging-related sex-steroid deficiency. We therefore hypothesize that an increased estrogen production is comparable to the estrogen-mimicking effects of Zip14^L438R^, resulting in aggravation of symptoms in (female) HCI patients.

Finally, we aimed at identifying possible downstream mechanisms or second messengers through which ZIP14 mediates its effects by osteoblasts. Zip14 was previously shown to play an important role in G-protein coupled receptor (GPCR)-mediated signaling by importing Zn into the cytosol and maintaining basal cAMP levels [15]. We detected a 5-fold increase in cAMP levels in Saos-2 cells transfected with ZIP14^L441R^. Cyclic AMP is a well-known second messenger for several hormones, like PTH/PTHrP [15, 31, 32]. However, Zip14^L438R^ expression in osteoblasts did not result in a PTH-mimicking skeletal phenotype *in vivo*, not to say that it led to a PTH-contrasting phenotype. In the literature, the G-protein-coupled estrogen receptor (GPER) is documented to act predominantly intracellularly and stimulate cAMP production, calcium mobilization and c-Src. GPER is described to play a role in the reproductive system, nervous system and neuroendocrinology, immune system, cardiovascular system, pancreatic function and glucose metabolism and bone growth and chondrocyte metabolism [36]. Remarkably, *Zip14*^*-/-*^ mice are characterized by impaired gluconeogenesis, hyperinsulinemic/diabetic pancreatic islets, chronic inflammation state, osteopenia and growth retardation [14, 15]. Next, since *Zip14*^*-/-*^ mice have a proinflammatory phenotype with increased systemic interleukin-6 (Il-6) levels that are coincident with a decrease in BMD [14], we also investigated NFAT signaling activity by ZIP14^L441R^. We demonstrated a doubled NFAT signaling activity in Saos-2 cells by ZIP14^L441R^. NFAT signaling in osteoblasts has been linked to the production of chemoattractants (TNF-α, IL-6) to attract osteoclast progenitors and hence increase osteoclast numbers, as seen in Zip14^L438R^ Ob-KI mice (with normal RANKL/OPG ratio). qRT-PCR analysis indeed confirmed a significant higher expression of *Il-6* and *Tnf* in osteoblasts derived from the long bones of Zip14^L438R^ Ob-KI mice, compared to long bone control osteoblasts. This difference in expression was not detected in calvarial osteoblasts, where no skeletal phenotype is present. We therefore believe that NFAT signaling and the production of inflammatory cytokines by Zip14^L438R^ in osteoblasts is also essential in the development of the skeletal pathology. Finally, GPER activation is also linked to increased intracellular calcium mobilization, which is known to bind activators of NFAT [36]. Our overall hypothesis therefore is that mutant Zip14 increases intracellular Zn levels, GPER signaling and cAMP-CREB and NFAT activity from the intracellular organelle where it resides, with estrogen-mimicking effects on osteoblasts.

Although we are convinced that we identified *ZIP14* as disease causing gene for HCI and a putative underlying pathological mechanism, a major unresolved question is the exclusive skull phenotype of these patients. Here, ZIP14, along with numerous other Zn transporters and Zn-dependent proteins, define a local and spatiotemporal micro-environment and, for some reason, only that of the internal cortex of HCI patients calvariae results in severe bone overgrowth. Whether this is due to a specific deficit in the skeletal cells of the calvariae or fortunate differences in the expression pattern of compensatory mechanisms in the rest of the skeleton, remains to be determined in the future by performing RNA sequencing and a proteomic analysis, for example.

## Materials and methods

### Patients

The family with HCI originates from The Netherlands and has been described in detail previously [1, 2, 4].

### Exome sequencing and gene identification

Peripheral blood was collected from 24 family members and five non-related partners. Genomic DNA was isolated from these blood samples using standard procedures.

Exome sequencing was performed on a female patient using the NimbleGen SeqCap EZ Human Exome V2 enrichment panel on the HiSeq2000 (Illumina Inc.). Data analysis was performed with DNA Nexus (DNAnexus Inc.; dnanexus.com). Variants were filtered for their absence in dbSNP and non-coding and synonymous variants were excluded. As published previously, we already defined a linkage region on chromosome 8 (chr8: 21,593,210–28,256,787). Variants present in this specific region were selected for further investigation.

Possible variants were confirmed with Sanger sequencing on other family members. Non-covered exons were amplified by GoTaq DNA polymerase-mediated PCR (Promega) with primers covering the exons and the intron-exon boundaries. Sequencing was carried out with the ABI 310 Genetic Analyser (Thermo Fisher Scientific), using an ABI Prism BigDye terminator cycle sequencing kit, version 1.1 (Thermo Fisher Scientific).

### Expression constructs and *in vitro* mutagenesis

Wildtype (WT) human full length *ZIP14* cDNA (NM_001128431.2) cloned in a pCMV6-XL6 vector was obtained from OriGene Technologies and the mutation (c.1322T>G, p.L441R ZIP14) was introduced using the QuickChange Site-Directed Mutagenesis Kit (Agilent Technologies). Similarly, a construct generating a truncated form of ZIP14 was created (p.W22X ZIP14). This construct is used as a negative control for transfection experiments.

Green fluorescent protein (GFP) fusion proteins for WT, mutant and truncated *hZIP14* were generated using the above described expression constructs as template. A PCR amplification was performed to disrupt the termination codon and create the correct restriction sites. Then, the complete region of interest was subcloned in a pEGFP-N1 vector (Clontech Laboratories). As a control, all cloned products were sequenced with Sanger sequencing.

### Subcellular localization

HEK293T cells were grown in DMEM medium with 10% FBS supplemented with 100 U/mL penicillin and 100 U/mL streptomycin (Life Technologies). Twenty-four hours prior to transfection, cells were plated at a density of 1 x 10^5^ cells/mL in 35mm glass bottom dishes coated with poly-D-lysine (MatTek Corporation). HEK293T cells were transfected with WT, L441R or W22X ZIP14-GFP constructs using Fugene 6 (Promega) in a 3:1 ratio (Fugene 6:DNA). As the mutation in HCI patients is dominant, a heterozygous model was created by co-transfecting WT and L441R ZIP14-GFP. Forty-eight hours after transfection, cells were fixed with methanol, washed with PBS (Thermo Fisher Scientific), incubated with UltraCruz Blocking Reagent (sc-516214, Santa Cruz Biotechnology) for 30 minutes and washed PBS. Specific staining of the Golgi apparatus and early and late endosomes was obtained by first using monoclonal IgG_1_ antibodies targeting golgin-97 (sc-59820, Santa Cruz Biotechnology, 1:300 dilution), EEA1 (sc-137130, Santa Cruz Biotechnology, 1:100 dilution) and Rab7 (sc-376362, Santa Cruz Biotechnology, 1:200 dilution), respectively. Then, after washing with PBS, a mouse IgG kappa binding protein (m-IgGκ BP) conjugated to CruzFluor 555 (sc-516177, Santa Cruz Biotechnology, 1:100 dilution) was used to provide a specific fluorescent signal. Fluorescent staining of the plasma membrane was performed by incubating the fixed HEK293T cells with 1μg/mL tetramethylrhodamine conjugate of wheat germ agglutinin (Thermo Fisher Scientific) for 10 minutes and washed with PBS. Vectashield antifade mounting medium with 4',6-diamidino-2-phenylindole (DAPI; Vector Laboratories) was used to preserve fluorescence and to stain the nucleus. High resolution images were obtained using an Eclipse Ti-E inverted microscope (Nikon) attached to a dual spinning disk confocal system (UltraVIEW VoX; PerkinElmer) equipped with 405, 488 and 561nm diode lasers for excitation of blue, green and red fluorophores, respectively. Images were acquired and processed using Volocity 6.0.1 software (PerkinElmer).

### Zinc transport

Uptake of ^65^Zn and accumulation of Zn^2+^ with FluoZin3-AM in HEK293T cells were performed as described before [10, 37, 38]. In short, for ^65^Zn-uptake, HEK293T cells were plated at a density of 5 x 10^5^ cells/mL and transiently transfected with the WT, L441R or W22X ZIP14 expression vector, using the Effectene Transfection Reagent (Qiagen). An empty vector was used as a transfection control. Forty-eight hours after transfection, cells were washed with HBSS (pH 7.0, Thermo Fisher Scientific) and incubated at 37°C in serum-free DMEM containing ^65^Zn (GE Healthcare) and 4μM ZnCl_2_ for 15 minutes. Cells were washed three times with wash buffer (0.9% NaCl, 10mM EDTA, 10mM HEPES) and then solubilized with 0.2% SDS and 0.2M NaOH for 1 hour. Uptake of ^65^Zn was measured with a γ-ray spectrometer. Total protein concentrations were measured with the Pierce BCA protein assay kit (Thermo Fisher Scientific) and used as a normalizer.

For Zn^2+^ accumulation, transfected HEK293T cells were incubated with 5μM FluoZin3-AM (Thermo Fisher Scientific) in serum-free DMEM for 30 minutes at 37°C. Cells were then stimulated with 40μM ZnCl_2_ after which fluorescence was measured at 494/516nm excitation/emission[37].

### Immunohistochemistry of bone tumor tissue

From the Tumorbank of the Antwerp University Hospital (Belgium), tissue of a giant cell tumor of bone and an osteoblastoma were obtained. Tissue specimens were fixed in 4% formaldehyde and paraffin embedded on a routine basis. Five μm-thick sections were subjected to heat-induced antigen retrieval by incubation in 10mM citrate buffer (pH 6.0) for 20 minutes at 97°C. Subsequently, endogenous peroxidase activity was quenched by incubating the slides in peroxidase blocking buffer (DAKO) for 10 minutes. Incubation with primary anti-human ZIP14 antibody (PA5-21077, Thermo Fisher Scientific, 1:200 dilution) was performed at room temperature for 1 hour. Bound antibody was detected with the Envision FLEX+ detection kit (DAKO) using 3,3’-diaminobenzidine chromogen solution (DAKO). A negative control, using a rabbit IgG isotype control (10500C, Thermo Fisher Scientific, 11.2ng/μL) was included in each staining run and did not show positive expression in osteoblasts or giant cells (S7 Fig). Sections were counterstained with haematoxylin, dehydrated and mounted.

### Expression of *Zip14* in KS483 cells and osteoclasts

KS483 cells, murine pre-osteoblast cells with mesenchymal characteristics, were used to examine the expression of murine *Zip14* (*mZip14*) during the differentiation to mature and mineralizing osteoblasts. KS483 cells were grown in α-MEM with GlutaMAX (Thermo Fisher Scientific) and 10% FBS (Lonza) supplemented with penicillin-streptomycin (Thermo Fisher Scientific). Cells were plated at a density of 2 x 10^4^ cells/mL in a 24-well plate and incubated at 37°C in humidified air containing 5% CO_2_. RNA was extracted at day 4, 7, 11, 14, 18, 21, 24 and 28 with the ReliaPrep RNA Cell Miniprep System (Promega) and reverse transcribed with an oligo-dT primer and Superscript II Reverse Transcriptase (Thermo Fisher Scientific). Quantitative real-time PCR (qPCR) analysis was performed on all samples with qPCR Core kit for SYBR Green I, No Rox (Eurogentec). For each sample, *mZip14* expression was analyzed and normalized to *b2m*, *rpl13a* and *ubc* expression. Stability of reference genes was verified using geNorm (Biogazelle) and efficiency of all primer pairs was checked with the qbase+ software (Biogazelle). Expression of target and reference genes was quantified using qbase+ software.

To assess expression of *mZip14* in osteoclasts, bone marrow cells from calvaria and long bones were isolated from mice as previously described [39]. Osteoclasts were cultured on plastic or bovine cortical bone slices with supplementation of M-CSF or M-CSF with RANKL. RNA from cultured bone marrow cells was isolated using the RNeasy Mini Kit (Qiagen) and reversed transcribed to cDNA for qPCR. Samples were normalized for the expression of *b2m* [39]. All primer sequences are available upon request.

### Histology of human skull biopsy samples

An occipital skull bone biopsy was taken during neurosurgical intervention from a 29-year old female patient with HCI, after receipt of informed consent by the patient. The biopsy specimen was fixed in 4% paraformaldehyde, decalcified and embedded in paraffin. Sections were stained by standard hematoxylin-eosin staining procedures. As a control sample, an occipital skull bone biopsy was taken during neurosurgical intervention from a 37-year old female with a posterior fossa meningioma, after receipt of informed consent. Peripheral blood was collected for the isolation of genomic DNA and genetic screening of *ZIP14* with Sanger sequencing. The biopsy specimen was fixed, decalcified, embedded and stained according to the same procedures as described above. Quantification of the number of Haversian channels and osteocytes was performed on three microscopic images of the patient and control externae/internae of the skull and of the patient vertebral cortex.

### *μ*CT of *Zip14*^*-/-*^ mice

Heterozygous Zip14 knockout (*Zip14*^*+/−*^) mice of the C57BL/6 strain were obtained from the Mutant Mouse Research Resource Consortium at the University of California, Davis via a contract. A breeding colony was established at the University of Florida, generating homozygous (*Zip14*^+/+^*)* WT and homozygous *Zip14* knockout (*Zip14*^*−/−*^) mice [13, 26]. *Zip14*^*-/-*^ (n = 7) and *Zip14*^*+/+*^ mice (n = 6) were fixed in 10% formalin and stored in 70% EtOH. μCT scans of the calvaria were generated with the SkyScan1076 system (Bruker microCT). Images were reconstructed with NRecon software and data were analyzed with Dataviewer and CTAn (Bruker microCT). Cortical thickness and porosity were measured at the calvariae. Nomenclature, symbols and units used are those recommended by the Nomenclature Committee of the American Society of Bone and Mineral Density[40].

### Generation of a mouse model for HCI

The mutated leucine at amino acid position 441 in ZIP14 of HCI patients is highly conserved in mice and corresponds to mL438 in both isoforms of mZip14 (NP_001128624.1; NP_659057.2). As no difference in function between both isoforms was reported, wildtype full length mZip14 cDNA corresponding to NP_001128624.1 cloned in a pCMV6-Entry vector was obtained from OriGene Technologies (MC216777). The mutation resulting in the p.L438R substitution was inserted using the QuickChange Site-Directed Mutagenesis kit (Agilent Technologies). This construct was sent to genOway (France) to create a mouse model with *Zip14*^*L438R*^ through targeted insertion within the *ROSA26* locus via homologous recombination in embryonic stem cells. A *lox*P-flanked transcriptional STOP cassette is incorporated between *Zip14*^*L438R*^ and a CAG promoter to allow the expression of *Zip14*^*L438R*^ to be dependent upon the Cre recombinase (S8 Fig). For breeding, Sox2-Cre mice, Runx2-Cre mice and CtsK-Cre mice were kindly provided by Vincent Timmerman and Delphine Bouhy [41] (University of Antwerp), Jan Tuckermann[28] (Universität Ulm) and Rachel Davey [27] (University of Canberra), respectively.

Mice homozygous for the floxed mutant *Zip14* allele (*Zip14*^*flox/flox*^) were crossed with the different Cre mice. Offspring was weaned after 3 weeks and marked by ear clipping. DNA, isolated from the tail tip, was used for genotyping of the *ROSA26* locus by performing two PCRs (S8 Fig). The Expand Long Template PCR System (Roche) and dNTP solution mix (Bio-Rad Laboratories) are used for both genotyping PCRs. Fragments were separated on a 2% agarose gel simultaneously running a GeneRuler 100bp Plus DNA Ladder and GeneRuler 1kb DNA Ladder (Thermo Fisher Scientific). In offspring from breedings with Runx2-Cre and CtsK-Cre mice, a third PCR is performed to check the corresponding Cre-allele. Here, standard GoTaq DNA polymerase-mediated PCR reactions (Promega) were performed.

Skeletal phenotyping was performed at the age of 6 months, corresponding to the age of 30 years in humans at which the HCI phenotype is prominent[42]. Since no gender-specific differences were found, only the data from male mice are presented in this manuscript. All mice were given two injections of 30 mg/kg calcein at 9 and 2 days before death to assess dynamic histomorphometric indices. At least six mice per group were subjected to histomorphometry and serum analysis to obtain sufficient results to perform statistical analyses. All mice were maintained on a twelve-hour light-dark cycle, with a regular unrestricted diet available *ad libitum*.

### Skeletal phenotyping of mice

Dissected skeletons were fixed in 3.7% PBS-buffered formaldehyde for 18 hours at 4°C and stored in 80% ethanol. All mice were analyzed by contact X-ray and μCT scanning. For the latter, a μCT 40 desktop cone-beam μCT (Scanco Medical) was used and reconstructed slices were examined using the Scanco MicroCT software suite. To assess biomechanical stability of the femora, three-point bending assays and a quantitative backscattered electron imaging (qBEI) analysis were performed as described[43–46]. The lumbar vertebral bodies (L1-L4) and one tibia were dehydrated in ascending alcohol concentrations and embedded in methylmethacrylate as previously described[46]. Parameters of structural and cellular histomorphometry were quantified on Von Kossa/Van Gieson and toluidine blue stained sections, respectively, of 4μm thickness. Analysis of bone volume, trabecular number, trabecular spacing, trabecular thickness, and the determination of osteoblast and osteoclast numbers and surface were carried out according to standardized protocols using the OsteoMeasure histomorphometry system (OsteoMetrics). Dynamic histomorphometry was performed on unstained 12μm sections of the vertebral bodies and tibia as previously described [46].

### Primary murine osteoblast cultures

Primary osteoblasts were isolated from calvaria and long bones (tibiae) of *Zip14*^*flox/-*^ and *Zip14*^*flox/-*^*; Runx2-Cre* mice as described previously [47]. In brief, cleaned calvariae and long bones were cut into small pieces and incubated with 2 mg/ml collagenase II (Sigma) solution for 2 h at 37°C in a shaking water bath. Then, the bone fragments were washed and cultured in α-MEM containing 10% FCS, 100 U/ml penicillin, 100 μg/ml streptomycin, and 250 ng/ml amphotericin B in 25 cm^2^ culture flasks. After confluence, we removed the bone fragments, the confluent layers were trypsinized and the cells were replated in 24-well plates for 21 days.

RNA of primary osteoblasts was isolated at day 0, day 14 and day 21 of differentiation using the RNeasy Mini Kit (Qiagen) and reverse transcribed to cDNA using the First Strand cDNA synthesis kit (Thermo-Fischer Scientific) for qPCR. qPCR reactions were performed in a 15 μl volume containing 2 ng cDNA, 7.5 μl SYBR Greener qPCR supermix (Invitrogen) and 300 nM of each primer [47]. Samples were normalized for the expression of *Hprt*.

Moreover, cDNA samples from day 0 calvarial and long bone osteoblasts were used for the amplification and sequencing of the region surrounding the c.1535 T>G (p.L438R) mutation in *Zip14*. Amplification was performed using a GoTaq2 polymerase-mediated PCR (Promega Corporation) and verified by agarose gel electrophoresis. Hereafter, primers and unincorporated dNTPs were removed using exonuclease I (New England Biolabs) and calf intestine alkaline phosphatase (CIAP, Roche Applied Science). Sequencing was carried out directly on purified fragments with the ABI 310 Genetic Analyzer (Applied Biosystems), using an ABI Prism BigDye terminator cycle sequencing ready reaction kit, version 1.1 (Applied Biosystems). The BigDye XTerminator purification kit was used as purification method for DNA sequencing with the purpose of removing unincorporated BigDye terminators.

### Biochemical assays

ELISA was used to determine serum concentrations of procollagen I C-terminal propeptide (PICP; SEA570Mu, USCN), C-terminal telopeptide (RatLaps (CTX-I) EIA, AC-06F1, Immunodiagnostic Systems), osteoprotegerin (OPG; MOP00, R&D Systems) and receptor activator of nuclear factor kappa-B ligand (RANKL; MTR00, R&D Systems).

### Luciferase reporter assays

HEK293T and Saos-2 cells were grown in DMEM (Thermo Fisher Scientific) supplemented with FBS (10% v/v). Twenty-four hours prior to transfection, cells were plated at 0.3 x 10^5^ cells/well in 96-well plates. Cells were transiently transfected with pRL-tK (2,5ng) and pCRE-Luc, NF-kB-Luc or pGL4.30 (NFAT-Luc, Promega) (25ng) along with 20ng of empty pcDNA3.1 vector, WT, L441R or W22X ZIP14 expression constructs using Fugene 6 (HEK293T cells) or ViaFect (Saos-2 cells) (Promega). Each transfection was carried out in triplicate and repeated independently in three separate experiments. Forty-eight hours after transfection, cells were lysed and firefly and renilla luciferase activity were measured on a Glomax Multi+ Luminometer (Turner Designs) using the dual luciferase reporter assay system (Promega). Finally, the ratio of the firefly and renilla luciferase measurement was calculated.

### Statistics

All data are presented as mean values ± SD and analyzed by a one-way ANOVA or a two-tailed Student’s t-test. Both statistical tests were provided by the SPSS v22.0 software (SPSS Inc). Statistical analysis of the mouse phenotyping data was performed by comparing the results of osteoblast knock-in mice and osteoclast knock-in mice with those of heterozygous Zip14^flox^ animals. Here, a value of *p*<0.05 (*) and *p*<0.025 (**) were considered statistically significant and significant after Bonferroni correction, respectively.

### Study approval

All HCI patients gave written informed consent, and the study was approved by the Committee of Medical Ethics of the University of Antwerp, according to the Declaration of Helsinki (EC UA 12/3/29). The skull biopsy specimen from an individual with a posterior fossa meningioma was obtained after receipt of informed consent and this study was approved by the Committee for Medical Ethics of the Antwerp University Hospital (EC UZA 16/14/166). All animal experiments were conducted according to the National Institutes of Health Guide for the Care and Use of Laboratory Animals and approved by the Committee of Medical Ethics of the University of Antwerp (ED 2012–01).

## Supporting information

S1 Fig Subcellular localization of wildtype and mutant (L441R) ZIP14 in the Golgi apparatus and in early and late endosomes of HEK293T cells. Red fluorescent staining of markers for the Golgi apparatus (golgin-97, left panel) and early (EEA1, central panel) and late endosomes (Rab7, right panel) was performed after transfection of a green fluorescent protein (GFP)-tagged wildtype or L441R ZIP14 in HEK293T cells. Merged figures demonstrate expression of wildtype and L441R ZIP14 in the Golgi apparatus and in early and late endosomes. Scale bars, 13μm.
(TIF)

S2 Fig μCT analysis of femora of controls (*Zip14*^*fl/-*^) and conditional Zip14^L438R^ female knock-in mice. **(A)** 3D reconstruction of whole femora of *Zip14*^*fl/-*^ controls, *Zip14*^*fl/-*^*; Runx2-Cre* and *Zip14*^*fl/-*^*; CtsK-Cre* mice. Femora of *Zip14*^*fl/-*^*; Runx2-Cre* mice show an increased cortical thickness and decreased midshaft diameter along with a decreased trabecular bone mass. **(B) μ**CT analysis of cortical (Ct) bone parameters confirms a significantly increased cortical thickness (Ct.Th) and decreased midshaft diameter (Ms.D) of *Zip14*^*fl/-*^*; Runx2-Cre* mice. *Zip14*^*fl/-*^*; CtsK-Cre* mice have an increased cortical porosity (Ct.Po). **(C) μ**CT analysis of trabecular (Tb) bone parameters demonstrates a lower, albeit not significantly, decreased trabecular bone volume (BV/TV), number (Tb.N), connecting density (Conn.D) and increased separation (Tb.Sp) in *Zip14*^*fl/-*^*; Runx2-Cre* mice. N = 3 animals/genotype; *: *p*<0.05; **: *p*<0.025 by 2-tailed Student’s t-test (compared to *Zip14*^*fl/-*^ mice).
(TIF)

S3 Fig Structural and cellular properties of the skeletal phenotype of conditional Zip14^L438R^ female knock-in mice. **(A)** Representative undecalcified spine (upper row) and tibia sections (bottom row) from *Zip14*^*fl/-*^, *Zip14*^*fl/-*^*; Runx2-Cre* and *Zip14*^*fl/-*^*; CtsK-Cre* mice stained with von Kossa/van Gieson. Vertebrae of *Zip14*^*fl/-*^*; Runx2-Cre* mice show less trabecular bone, whereas tibiae of these mice show an increased cortical thickness and decreased midshaft diameter compared to *Zip14*^*fl/-*^ controls. **(B)** Quantitative analysis of trabecular (Tb) bone parameters on lumbar spine sections stained with Von Kossa/Van Gieson confirms a significantly decreased trabecular bone volume (BV/TV) and number (Tb.N) in *Zip14*^*fl/-*^*; Runx2-Cre* mice, whereas trabecular BV/TV, Tb.N and trabecular thickness (Tb.Th) are increased in *Zip14*^*fl/-*^*; CtsK-Cre* mice. **(C)** Quantification of the bone surface covered by osteoblasts (Ob.S/BS), osteoblast number per bone perimeter (N.Ob/B.Pm), osteoclast surface per bone surface (Oc.S/BS) and osteoclast number per bone perimeter (N.Oc/B.Pm) in the vertebral bodies analyzed using toluidine blue staining. Both Oc.S and N.Oc are significantly increased in female *Zip14*^*fl/-*^*; Runx2-Cre* mice. N = 3 animals/genotype; *: *p*<0.05; **: *p*<0.025 by 2-tailed Student’s t-test (compared to *Zip14*^*fl/-*^ mice).
(TIF)

S4 Fig Dynamic histomorphometric analysis of conditional Zip14^L438R^ female knock-in mice. **(A)** Dynamic histomorphometry of the tibial endocortical (Ct), **(B)** tibial periosteal (P) and **(C)** trabecular (Tb) bone surface measuring the mineralizing surface (MS/BS), bone formation rate (BFR/BS) and mineral apposition rate (MAR) in *Zip14*^*fl/-*^*; Runx2-Cre* and *Zip14*^*fl/-*^*; CtsK-Cre* mice. N = 3 animals/genotype.
(TIF)

S5 Fig Quantitative structural histomorphometry of spine sections from controls (*Zip14*^*fl/-*^) and conditional Zip14^L438R^ knock-in mice. Quantitative analysis of trabecular (Tb) bone parameters on lumbar spine sections stained with Von Kossa/Van Gieson confirms a significantly decreased trabecular bone volume (BV/TV), number (Tb.N), and increased separation (Tb.Sp) in *Zip14*^*fl/-*^*; Runx2-Cre* mice. N = 6 animals/genotype; *: *p*<0.05; **: *p*<0.025 by 2-tailed Student’s t test (compared to *Zip14*^*fl/-*^ mice).
(TIF)

S6 Fig Dynamic histomorphometric analysis of the tibial periosteal (P) bone surface of conditional Zip14^L438R^ male knock-in mice. Dynamic histomorphometry of the tibial periosteal (P) bone surface indicates. N = 6 animals/genotype; *: *p*<0.05; **: *p*<0.025 by 2-tailed Student’s t-test (compared to *Zip14*^*fl/-*^ mice).
(TIF)

S7 Fig Negative control for immunohistochemistry experiments. Immunohistochemistry of osteoblastoma and giant cell tumor tissue with a rabbit IgG isotype control shows no positive signal in osteoblasts (black line), in giant osteoclast-like cells (arrowheads) and in osteocytes. Scale bars upper figures, 500μm; scale bars lower figures, 100μm.
(TIF)

S8 Fig Generation and genotyping of a floxed Zip14^L438R^ mouse model. **(A)** A mouse model with floxed Zip14^L438R^ was generated through targeted insertion within the ROSA26 locus. A loxP-flanked transcriptional STOP cassette is incorporated between Zip14^L438R^ and its CAG promoter to allow the expression of the resulting transgene to be dependent upon the Cre recombinase. **(B)** A first PCR for genotyping (left) is to detect the Zip14^flox^ and Cre-mediated excised (Zip14^L438R^) locus, with amplicons of 3428bp and 410bp in size, respectively, whereas the wildtype allele gives no amplification. A second PCR (right) is performed to distinguish homozygous Zip14^flox/flox^ (998bp), heterozygous Zip14^flox/-^ or Zip14^L438R/-^ (998bp + 304bp) and homozygous wildtype (304bp) mice. **(C)** Sanger sequencing was performed to verify *Zip14*^*L438R*^ (c.1535 T>G) overexpression in cDNA of primary osteoblasts derived from calvariae and long bones of *Zip14*^*fl/-*^*; Runx2-Cre* mice. As these mice also express endogenous *Zip14*, a low wildtype (T-base) signal can be noted in both osteoblast types as well. **(D)** Femoral length and body weight of 6-month old *Zip14*^*fl/-*^ *controls*, *Zip14*^*fl/-*^*; Runx2-Cre* and *Zip14*^*fl/-*^*; CtskK-Cre* mice. N = 6 animals/genotype.
(TIF)
