# Supplementary figures and images for: Conditional mouse models support the role of SLC39A14 (ZIP14) in Hyperostosis Cranialis Interna and in bone homeostasis
Source: PLoS Genet. 2018 Apr 5;14(4):e1007321. doi: 10.1371/journal.pgen.1007321 (PMC5903675; doi:10.1371/journal.pgen.1007321)

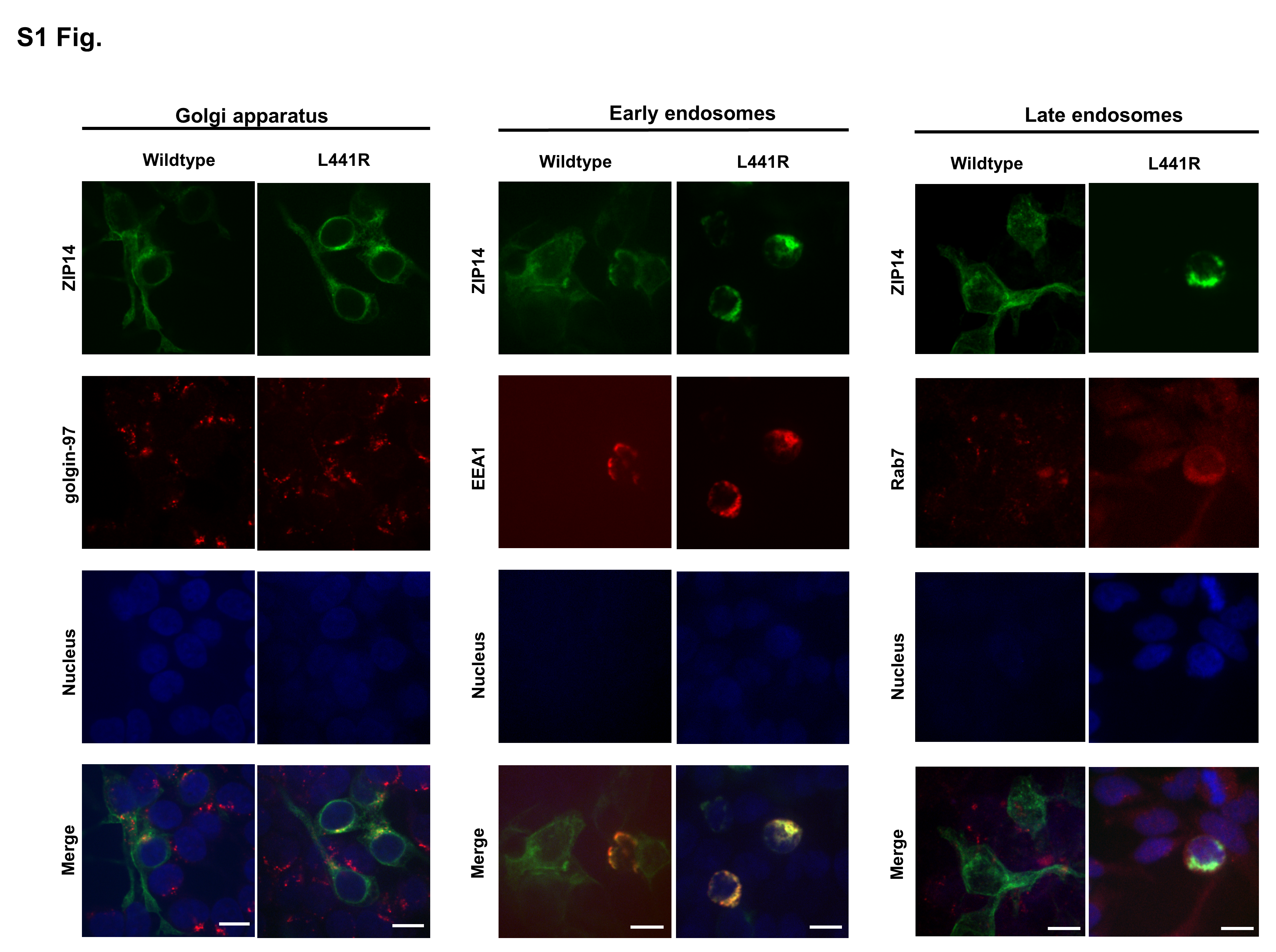

Supplement: S1 Fig — Red fluorescent staining of markers for the Golgi apparatus (golgin-97, left panel) and early (EEA1, central panel) and late endosomes (Rab7, right panel) was performed after transfection of a green fluorescent protein (GFP)-tagged wildtype or L441R ZIP14 in HEK293T cells. Merged figures demonstrate expression of wildtype and L441R ZIP14 in the Golgi apparatus and in early and late endosomes. Scale bars, 13μm. (TIF) [file pgen.1007321.s001.tif]

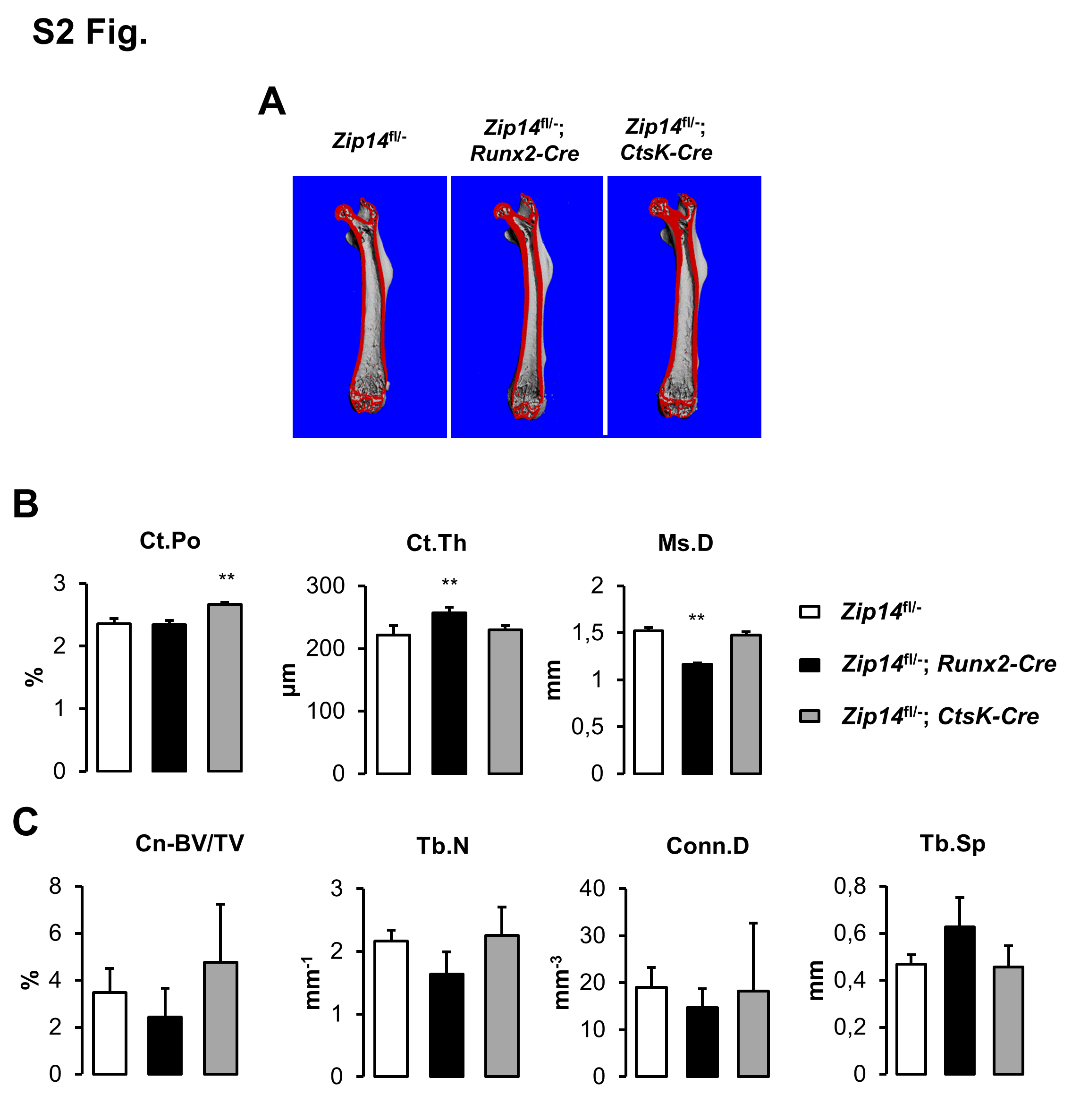

Supplement: S2 Fig — (A) 3D reconstruction of whole femora of Zip14fl/- controls, Zip14fl/-; Runx2-Cre and Zip14fl/-; CtsK-Cre mice. Femora of Zip14fl/-; Runx2-Cre mice show an increased cortical thickness and decreased midshaft diameter along with a decreased trabecular bone mass. (B) μCT analysis of cortical (Ct) bone parameters confirms a significantly increased cortical thickness (Ct.Th) and decreased midshaft diameter (Ms.D) of Zip14fl/-; Runx2-Cre mice. Zip14fl/-; CtsK-Cre mice have an increased cortical porosity (Ct.Po). (C) μCT analysis of trabecular (Tb) bone parameters demonstrates a lower, albeit not significantly, decreased trabecular bone volume (BV/TV), number (Tb.N), connecting density (Conn.D) and increased separation (Tb.Sp) in Zip14fl/-; Runx2-Cre mice. N = 3 animals/genotype; *: p<0.05; **: p<0.025 by 2-tailed Student’s t-test (compared to Zip14fl/- mice). (TIF) [file pgen.1007321.s002.tif]

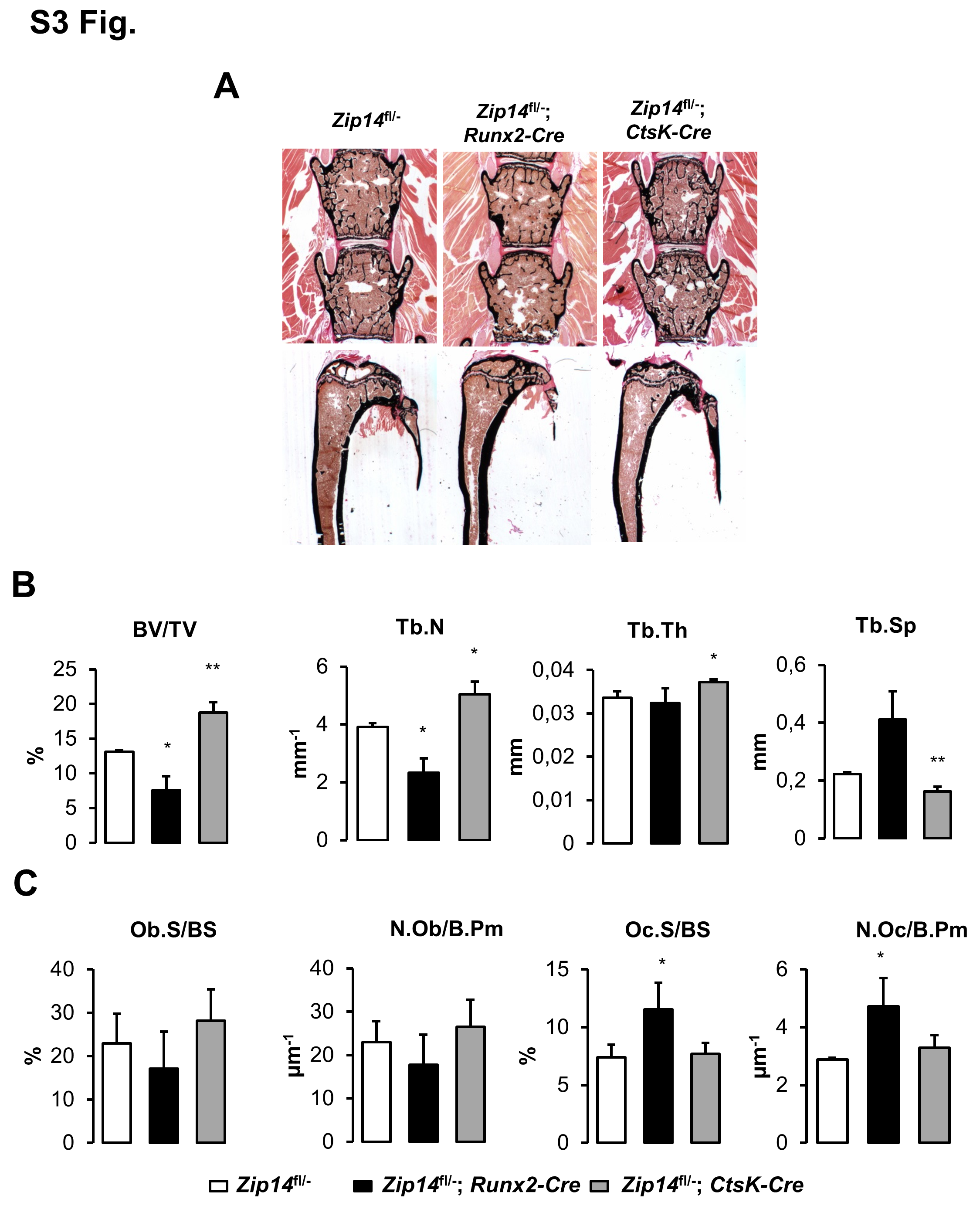

Supplement: S3 Fig — (A) Representative undecalcified spine (upper row) and tibia sections (bottom row) from Zip14fl/-, Zip14fl/-; Runx2-Cre and Zip14fl/-; CtsK-Cre mice stained with von Kossa/van Gieson. Vertebrae of Zip14fl/-; Runx2-Cre mice show less trabecular bone, whereas tibiae of these mice show an increased cortical thickness and decreased midshaft diameter compared to Zip14fl/- controls. (B) Quantitative analysis of trabecular (Tb) bone parameters on lumbar spine sections stained with Von Kossa/Van Gieson confirms a significantly decreased trabecular bone volume (BV/TV) and number (Tb.N) in Zip14fl/-; Runx2-Cre mice, whereas trabecular BV/TV, Tb.N and trabecular thickness (Tb.Th) are increased in Zip14fl/-; CtsK-Cre mice. (C) Quantification of the bone surface covered by osteoblasts (Ob.S/BS), osteoblast number per bone perimeter (N.Ob/B.Pm), osteoclast surface per bone surface (Oc.S/BS) and osteoclast number per bone perimeter (N.Oc/B.Pm) in the vertebral bodies analyzed using toluidine blue staining. Both Oc.S and N.Oc are significantly increased in female Zip14fl/-; Runx2-Cre mice. N = 3 animals/genotype; *: p<0.05; **: p<0.025 by 2-tailed Student’s t-test (compared to Zip14fl/- mice). (TIF) [file pgen.1007321.s003.tif]

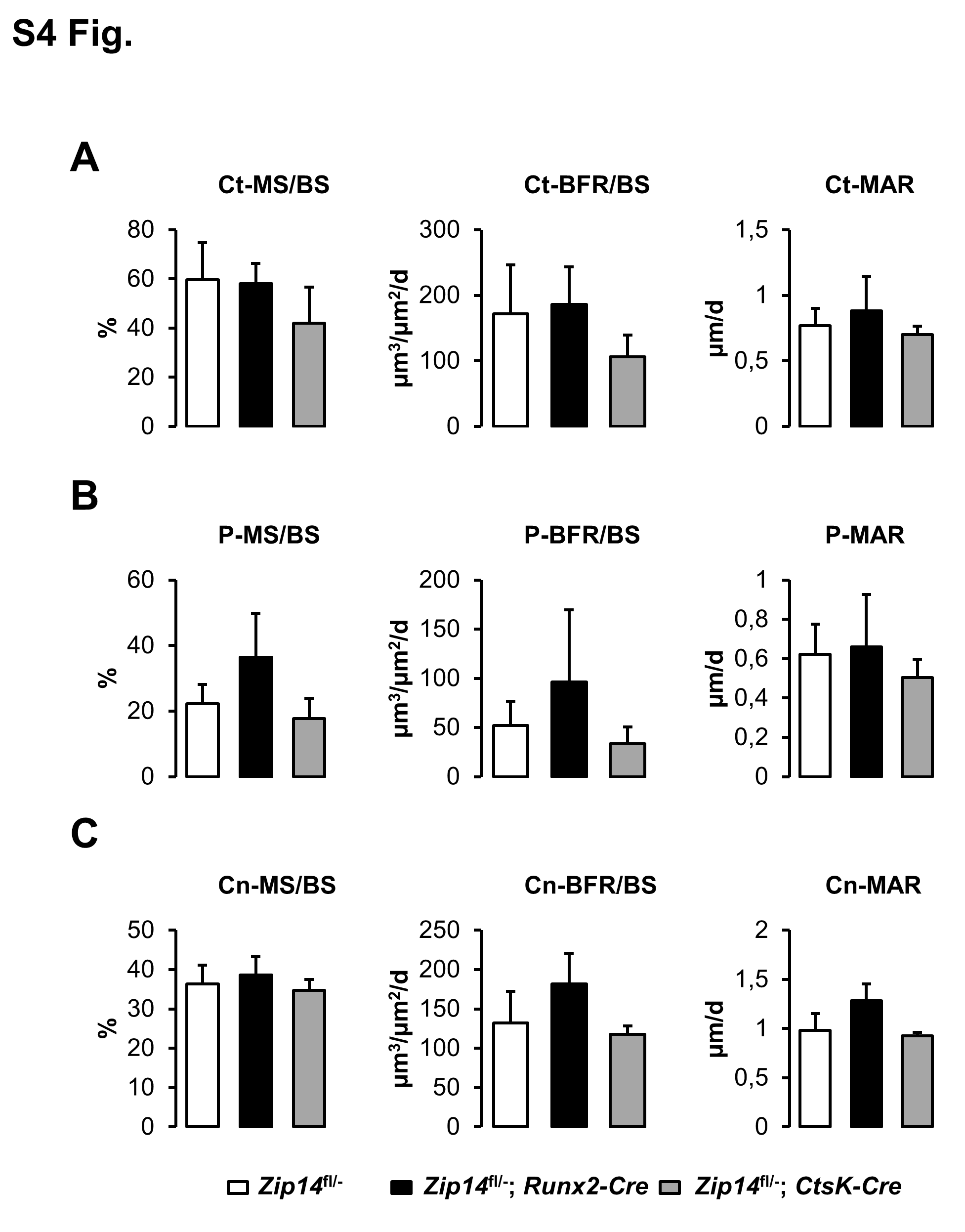

Supplement: S4 Fig — (A) Dynamic histomorphometry of the tibial endocortical (Ct), (B) tibial periosteal (P) and (C) trabecular (Tb) bone surface measuring the mineralizing surface (MS/BS), bone formation rate (BFR/BS) and mineral apposition rate (MAR) in Zip14fl/-; Runx2-Cre and Zip14fl/-; CtsK-Cre mice. N = 3 animals/genotype. (TIF) [file pgen.1007321.s004.tif]

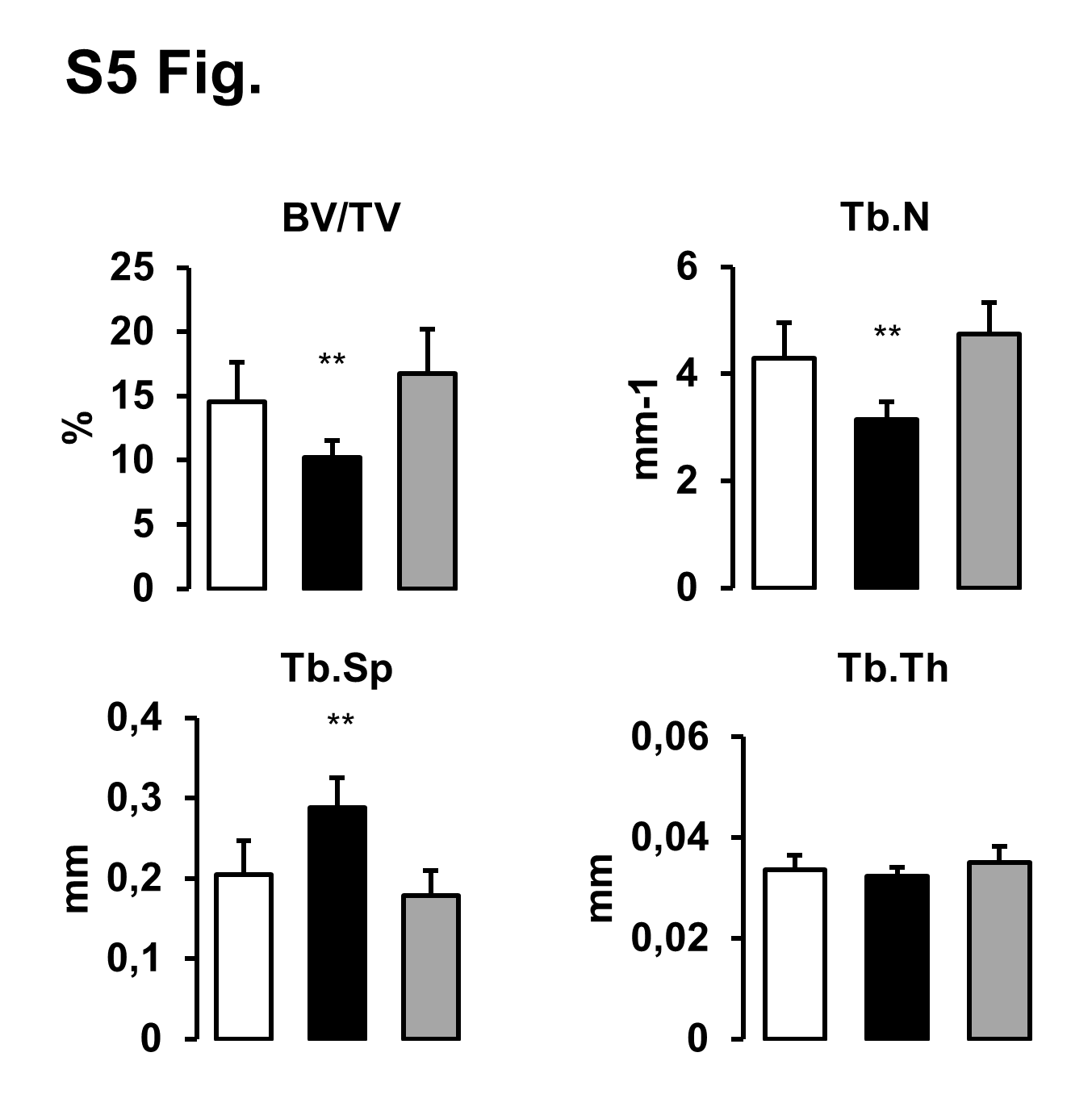

Supplement: S5 Fig — Quantitative analysis of trabecular (Tb) bone parameters on lumbar spine sections stained with Von Kossa/Van Gieson confirms a significantly decreased trabecular bone volume (BV/TV), number (Tb.N), and increased separation (Tb.Sp) in Zip14fl/-; Runx2-Cre mice. N = 6 animals/genotype; *: p<0.05; **: p<0.025 by 2-tailed Student’s t test (compared to Zip14fl/- mice). (TIF) [file pgen.1007321.s005.tif]

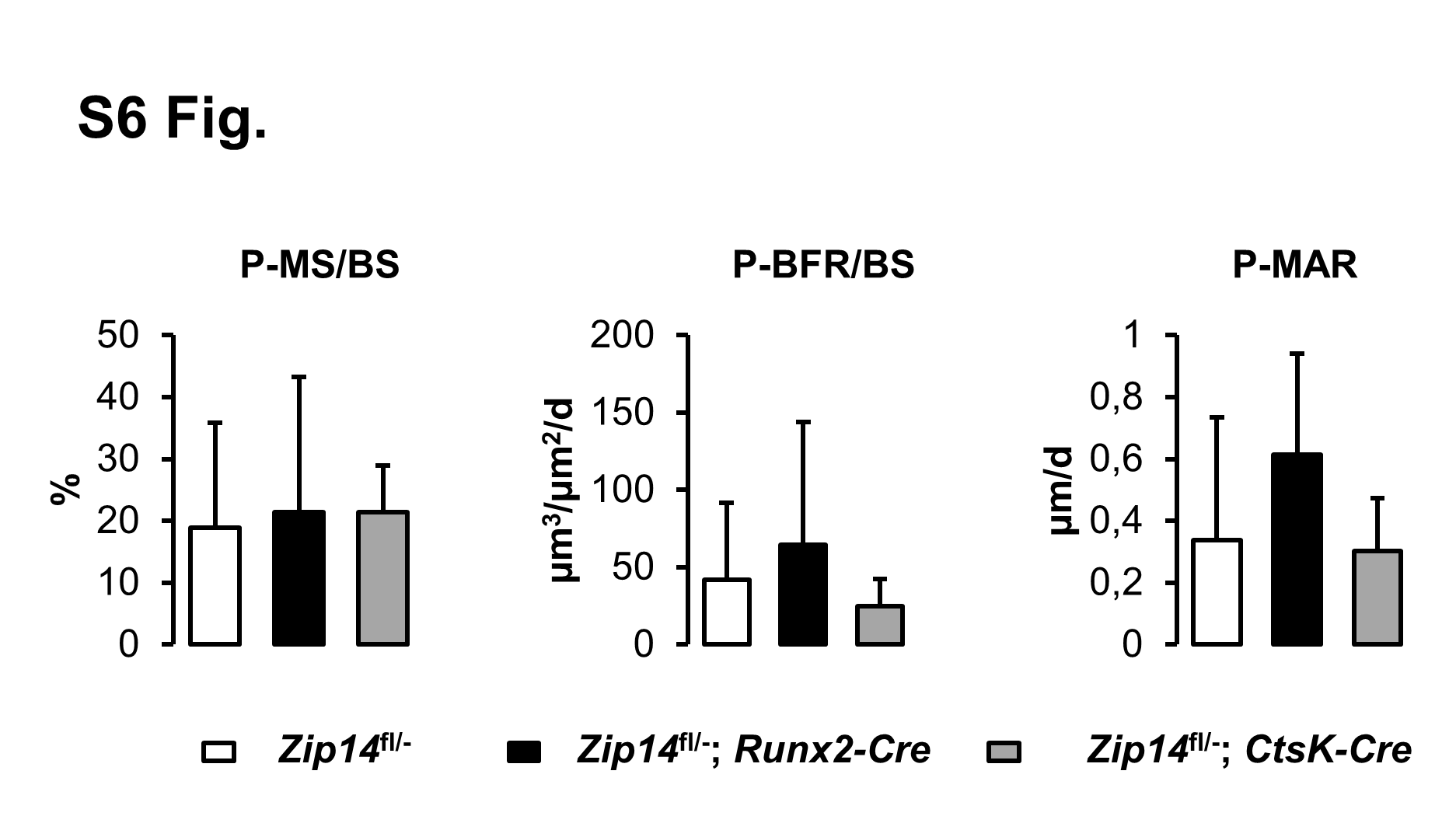

Supplement: S6 Fig — Dynamic histomorphometry of the tibial periosteal (P) bone surface indicates. N = 6 animals/genotype; *: p<0.05; **: p<0.025 by 2-tailed Student’s t-test (compared to Zip14fl/- mice). (TIF) [file pgen.1007321.s006.tif]

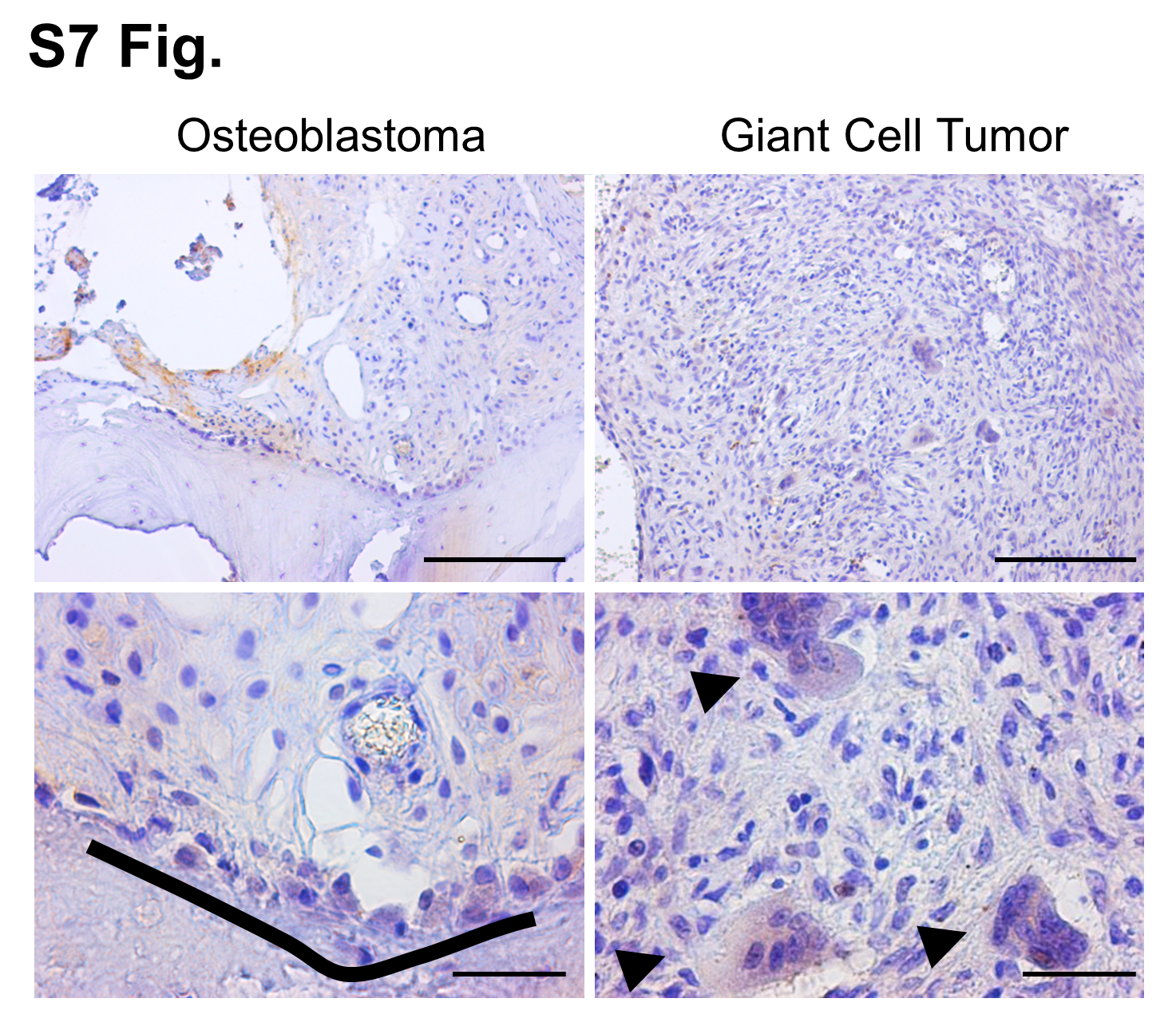

Supplement: S7 Fig — Immunohistochemistry of osteoblastoma and giant cell tumor tissue with a rabbit IgG isotype control shows no positive signal in osteoblasts (black line), in giant osteoclast-like cells (arrowheads) and in osteocytes. Scale bars upper figures, 500μm; scale bars lower figures, 100μm. (TIF) [file pgen.1007321.s007.tif]

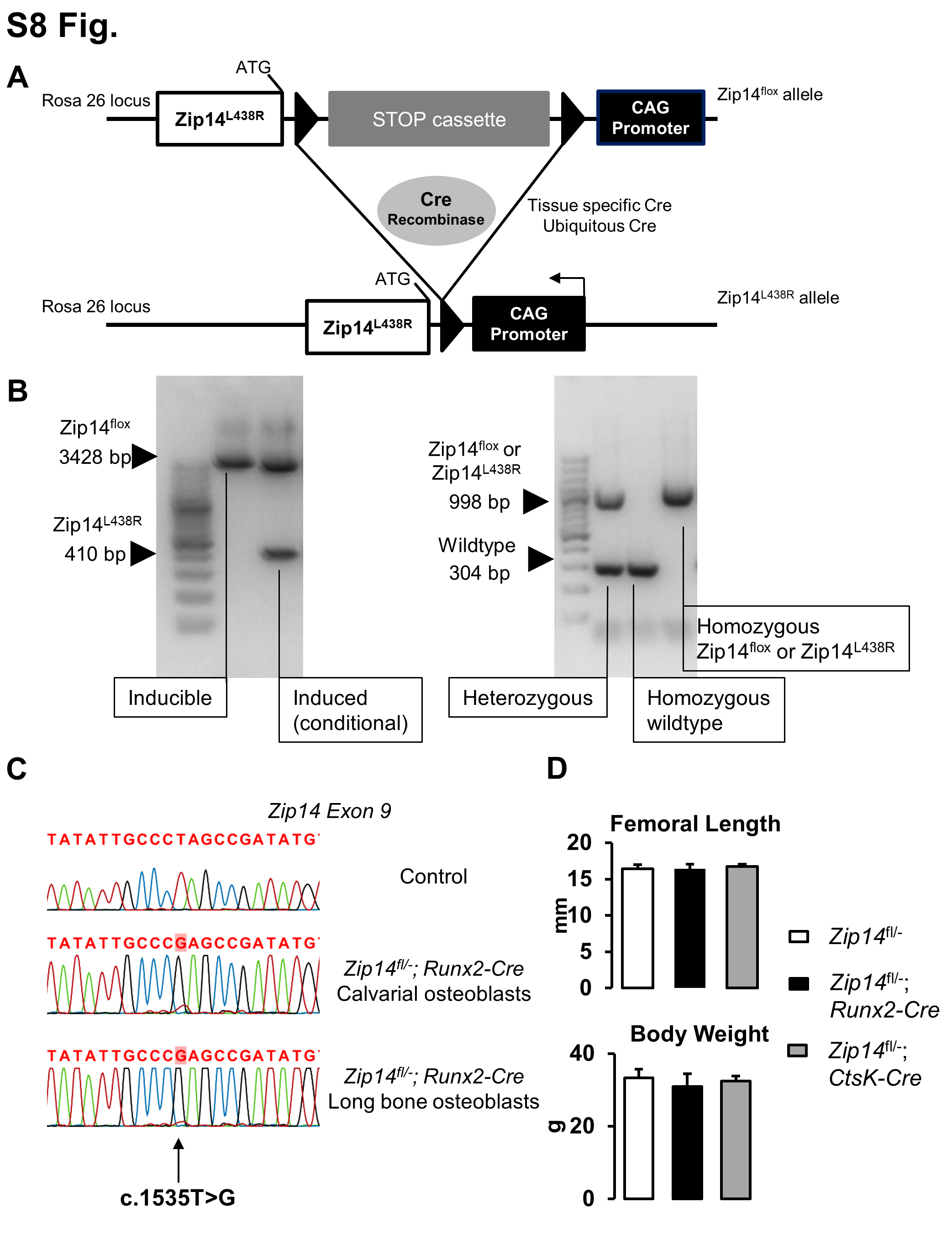

Supplement: S8 Fig — (A) A mouse model with floxed Zip14L438R was generated through targeted insertion within the ROSA26 locus. A loxP-flanked transcriptional STOP cassette is incorporated between Zip14L438R and its CAG promoter to allow the expression of the resulting transgene to be dependent upon the Cre recombinase. (B) A first PCR for genotyping (left) is to detect the Zip14flox and Cre-mediated excised (Zip14L438R) locus, with amplicons of 3428bp and 410bp in size, respectively, whereas the wildtype allele gives no amplification. A second PCR (right) is performed to distinguish homozygous Zip14flox/flox (998bp), heterozygous Zip14flox/- or Zip14L438R/- (998bp + 304bp) and homozygous wildtype (304bp) mice. (C) Sanger sequencing was performed to verify Zip14L438R (c.1535 T>G) overexpression in cDNA of primary osteoblasts derived from calvariae and long bones of Zip14fl/-; Runx2-Cre mice. As these mice also express endogenous Zip14, a low wildtype (T-base) signal can be noted in both osteoblast types as well. (D) Femoral length and body weight of 6-month old Zip14fl/- controls, Zip14fl/-; Runx2-Cre and Zip14fl/-; CtskK-Cre mice. N = 6 animals/genotype. (TIF) [file pgen.1007321.s008.tif]
